# Glycogen synthase protects neurons from cytotoxicity of mutant huntingtin by enhancing the autophagy flux

**DOI:** 10.1038/s41419-017-0190-5

**Published:** 2018-02-08

**Authors:** Anupama Rai, Pankaj Kumar Singh, Virender Singh, Vipendra Kumar, Rohit Mishra, Ashwani Kumar Thakur, Anita Mahadevan, Susarla Krishna Shankar, Nihar Ranjan Jana, Subramaniam Ganesh

**Affiliations:** 1 Department of Biological Sciences and Bioengineering, Indian Institute of Technology, Kanpur, 208016 India; 2 National Brain Research Centre, Manesar, 122051 India; 3 Department of Neuropathology, National Institute of Mental Health and Neuroscience, Bengaluru, 560029 India; 4 Present Address: Institut de Génétique et de Biologie Moléculaire et Cellulare (IGBMC), Illkirch, France

## Abstract

Healthy neurons do not store glycogen while they do possess the machinery for the glycogen synthesis albeit at an inactive state. Neurons in the degenerating brain, however, are known to accumulate glycogen, although its significance was not well understood. Emerging reports present contrasting views on neuronal glycogen synthesis; a few reports demonstrate a neurotoxic effect of glycogen while a few others suggest glycogen to be neuroprotective. Thus, the specific role of glycogen and glycogen synthase in neuronal physiology is largely unexplored. Using cellular and animal models of Huntington’s disease, we show here that the overexpression of cytotoxic mutant huntingtin protein induces glycogen synthesis in the neurons by activating glycogen synthase and the overexpressed glycogen synthase protected neurons from the cytotoxicity of the mutant huntingtin. Exposure of neuronal cells to proteasomal blockade and oxidative stress also activate glycogen synthase to induce glycogen synthesis and to protect against stress-induced neuronal death. We show that the glycogen synthase plays an essential and inductive role in the neuronal autophagic flux, and helps in clearing the cytotoxic huntingtin aggregate. We also show that the increased neuronal glycogen inhibits the aggregation of mutant huntingtin, and thus could directly contribute to its clearance. Finally, we demonstrate that excessive autophagy flux is the molecular basis of cell death caused by the activation of glycogen synthase in unstressed neurons. Taken together, our results thus provide a novel function for glycogen synthase in proteolytic processes and offer insight into the role of glycogen synthase and glycogen in both survival and death of the neurons.

## Introduction

In the animal kingdom, glycogen is the principal storage form of energy in all cell types except neurons as they store a negligible amount of glycogen^1^. Intriguingly, however, neurons are known to possess required machinery for the glycogen synthesis^2^, and the affected neurons in patients with Alzheimer’s disease, Parkinson’s disease, amyotrophic lateral sclerosis, or Lafora disease are also known to accumulate either the normal or an abnormal form of glycogen^3–5^. Although the exact reason for the increased glycogen in such neurons is not known, it is suggested that neurons resort to “storing” glycogen as a protective mechanism. For example, the glycogen synthesis enhanced in neurons under the conditions of hypoxia and endoplasmic reticulum stress is shown to have protective role^6,7^. A contrarian view is that the glycogen is neurotoxic and that the glycogen accumulation could possibly be the trigger for the neurodegenerative changes observed in the disorders^2^. Indeed, forced synthesis of glycogen in neurons lead to neurodegeneration, and enhanced brain aging in the mouse and *Drosophila* models^8^ supporting the aforementioned notion that glycogen could be neurotoxic.

One of the common underlying pathologies in the aforementioned neurodegenerative conditions is the presence of proteinaceous inclusions, which represent the aggregated misfolded/unfolded proteins in the affected neurons^9,10^. The aggregation might result from the increased production of abnormal proteins, such as the cytotoxic mutant version of disease linked protein, or due to a compromise in the protein quality control system due to defects in this pathway^9,10^. In either case, insufficient protection exerted by the proteostatic processes is thought to underlie the neurodegeneration^10^. Given the aforementioned observations, we hypothesized that glycogen is toxic to healthy neurons, and might be synthesized due to a protective mechanism induced in the neurons under physiological stress, including compromised proteostasis. Using cellular and animal models of Huntington disease, we show here that the cytotoxic mutant huntingtin induces neuronal glycogen synthesis and that the increased glycogen protects neurons by suppressing the aggregation of mutant huntingtin. We further show that glycogen synthase (GS) enzyme regulates autophagy flux and thus in active state help clear the aggregate load of the cell. We also show that overexpression of glycogen synthesizing proteins, induces autophagy in neurons and that the excessive autophagy is the cause of the death when neurons are not under physiological stress. Our results thus provide a novel function for GS in proteolytic processes and offer insight into the role of GS in both survival and death of neurons. We also demonstrate that the glycogen thus formed might aid in clearing the aggregate load by directly interacting with the protein and inhibiting the aggregation kinetics.

## Results

### Increased glycogen levels in cellular and animal models of Huntington disease

**Fig. 1:**
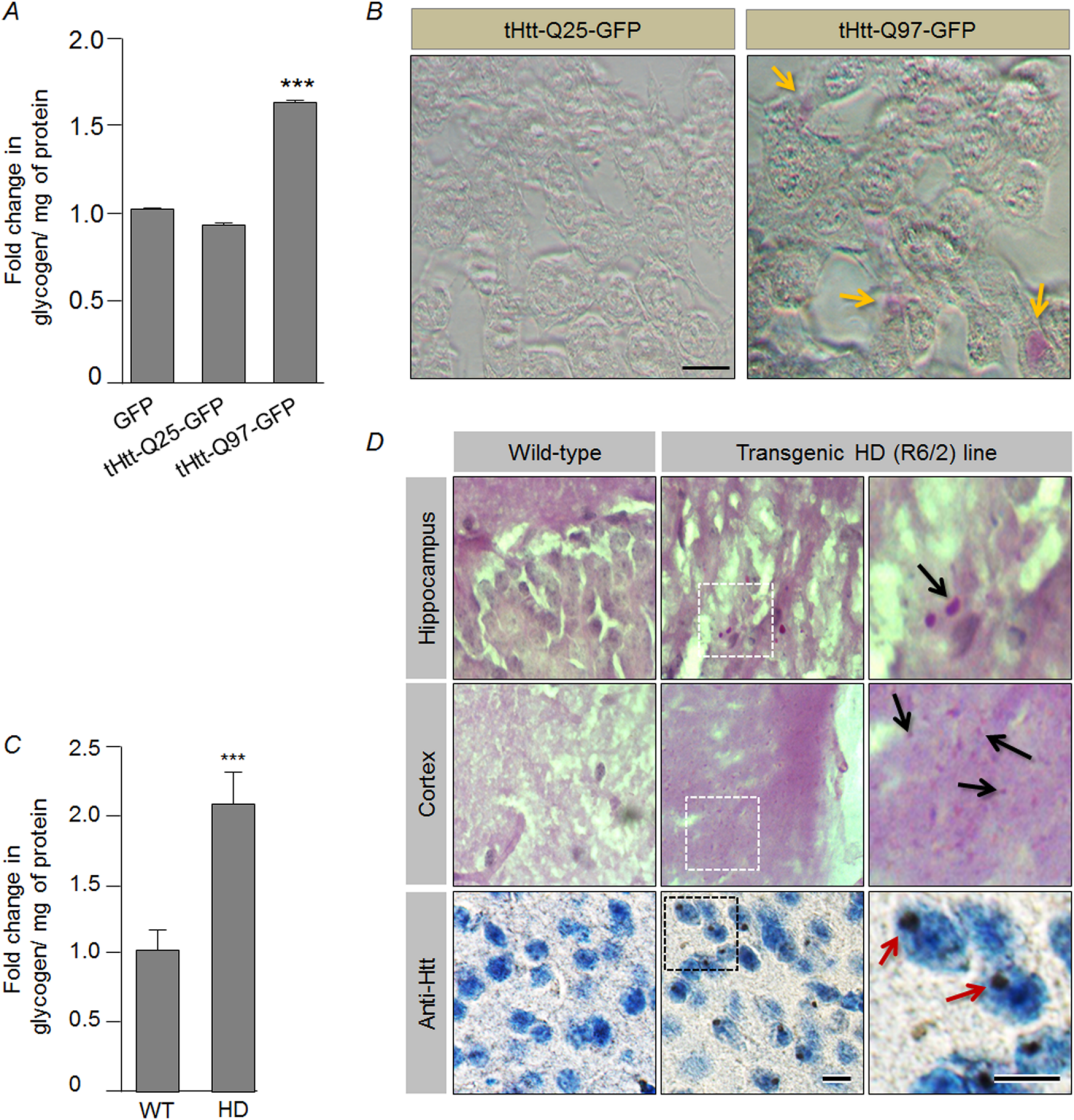
Increased glycogen levels in a neuronal cell and animal model of Huntington’s disease. **a** Bar diagram showing the fold change in the glycogen level (normalized to protein content) in Neuro2A cells transiently expressing the GFP, tHtt-Q25-GFP, or the tHtt-Q97-GFP as indicated. For calculating the change, the glycogen level measured in GFP-expressing cells was considered as 1 (*N* = 6; *t*-test; ***p ≤ 0.001). **b** Bright field images showing glycogen granules (identified by yellow arrows), as revealed by PAS staining, in Neuro2A cells transiently expressing the tHtt-Q97-GFP but not in those that express the tHtt-Q25-GFP (scale bar, 10 µm). **c** Bar diagram showing the fold change in the glycogen level (normalized to protein content) in the brain tissues (frontal cortex) of the R6/2 transgenic mouse as compared with the brain tissues (frontal cortex) of age-matched wild-type mouse. Fold change was measured by considering the value obtained for the wild-type sample as 1 (*N* = 3 for wild-type and 6 for R6/2; *t*-test; ***p ≤ 0.001). **d** Bright field images showing PAS-positive glycogen granules (upper two panels) (identified by black arrow) in the hippocampal and cortical regions of the brain regions of the R6/2 transgenic mouse, and the absence of such staining in the wild-type mouse. The lower panel reveals the huntingtin aggregates (identified by red arrow) in the transgenic mouse brain, as detected by immunostaining using anti-huntingtin antibody. Hematoxylin was used to counterstain the nuclei (scale bar, 10 µm)

We reasoned that the neuronal glycogen synthesis could be a stress response mechanism and tested the same using two neuronal cell lines of murine origin: Neuro2A and HT-22^11^. We found that the exposure of neuronal cells to hydrogen peroxide (oxidative stress) and MG132 (proteasomal stress) led to a significant increase in the glycogen levels as compared with the control set (see Supplementary Fig. S1A,B). Oxidative stress being one of the major stressors implicated in Huntington’s disease (HD)^12^, we next explored if cell and animal models of HD would show an increase in the neuronal glycogen level. For this, we transiently overexpressed expression constructs coding for the truncated huntingtin protein with either normal range of glutamine repeat (tHtt-Q25-GFP) or the expanded, disease-associated glutamine repeat tract (tHtt-Q97-GFP), of which the disease-associated mutant version (tHtt-Q97-GFP) is known to be cytotoxic^13,14^. Neuro2A cell lines transiently expressing the tHtt-Q97-GFP construct showed a significant increase in the glycogen content as compared with the cells expressing GFP or the tHtt-Q25-GFP construct (Fig. 1a). This observation was further strengthened by using conventional periodic acid–Schiff (PAS) staining, which revealed glycogen granules in Neuro2a cells transfected with the tHtt-Q97-GFP construct, but no visible staining in the cells expressing the tHtt-Q25-GFP construct was observed (Fig. 1b). Similarly, immunostaining using specific antibody against glycogen^15^ revealed larger glycogen particles and the 4′,6-diamidino-2-phenylindole (DAPI) staining demarcates the nuclear portion, thus revealing the presence of cytoplasmic larger glycogen particles exclusively in the tHtt-Q97-GFP-expressing cells (Supplementary Fig. S2). Intriguingly, a non-neuronal cell line, COS-7, did not show any difference in the glycogen level upon overexpression of the tHtt-Q97-GFP (Supplementary Fig. S1C), suggesting that stress-induced glycogen synthesis could be unique to neuronal cells. We further strengthened our hypothesis by estimating glycogen in Neuro2A cells by expressing tHtt-Q97-GFP along with SOD1, which is known to reduce oxidative stress when overexpressed^16^ and observed a reduction in the glycogen level as compared with the cells that expressed only the tHtt-Q97-GFP (see Supplementary Fig. S1D).

We next wanted to test if the animal model for HD would also show increased glycogen levels in the neurons. For this, we used the mouse model of HD, the R6/2 transgenic line^17^, expressing the mutant huntingtin with expanded polyglutamine tract, and known to show many of the HD pathologies including the ataxia, huntingtin aggregates, neurodegeneration^18^, and oxidative stress^12^ (Supplementary Fig. S1E). Similar to the cellular model of HD, a twofold increase in the glycogen was observed in the brain tissues of 2–3 months old HD mice as compared with their age-matched wild-type controls (Fig. 1c). Corroborating the biochemical data, PAS staining also showed glycogen accumulation in the brain tissues of HD mice (Fig. 1d).

### Activation of GS could underlie the increased glycogen content in the cellular model of HD

The increased glycogen levels observed in the HD models could either be due to an enhanced glycogen synthetic process or due to the suppression of the glycogen breakdown process. Glucose starvation is known to promote glycogen breakdown in cells^19^, and glucose starvation (6 h) did result in the reduction of the glycogen level in Neuro2A cells expressing tHtt-Q97-GFP, suggesting that glycogen breakdown is not altered in the cellular model of HD (Supplementary Fig. S3A). To test if the increased glycogen level is because of its increased synthesis, cytochalasin B, a competitive inhibitor of glucose uptake^20^ was added to the cells to inhibit glucose-dependent glycogen synthesis. The cytochalasin B treatment led to a significant reduction in the glycogen levels in the cell expressing the mutant huntingtin (Supplementary Fig. S3B), suggesting that the increased glycogen seen in Neuro2a cells expressing mutant huntingtin could be due to its enhanced synthesis.

**Fig. 2:**
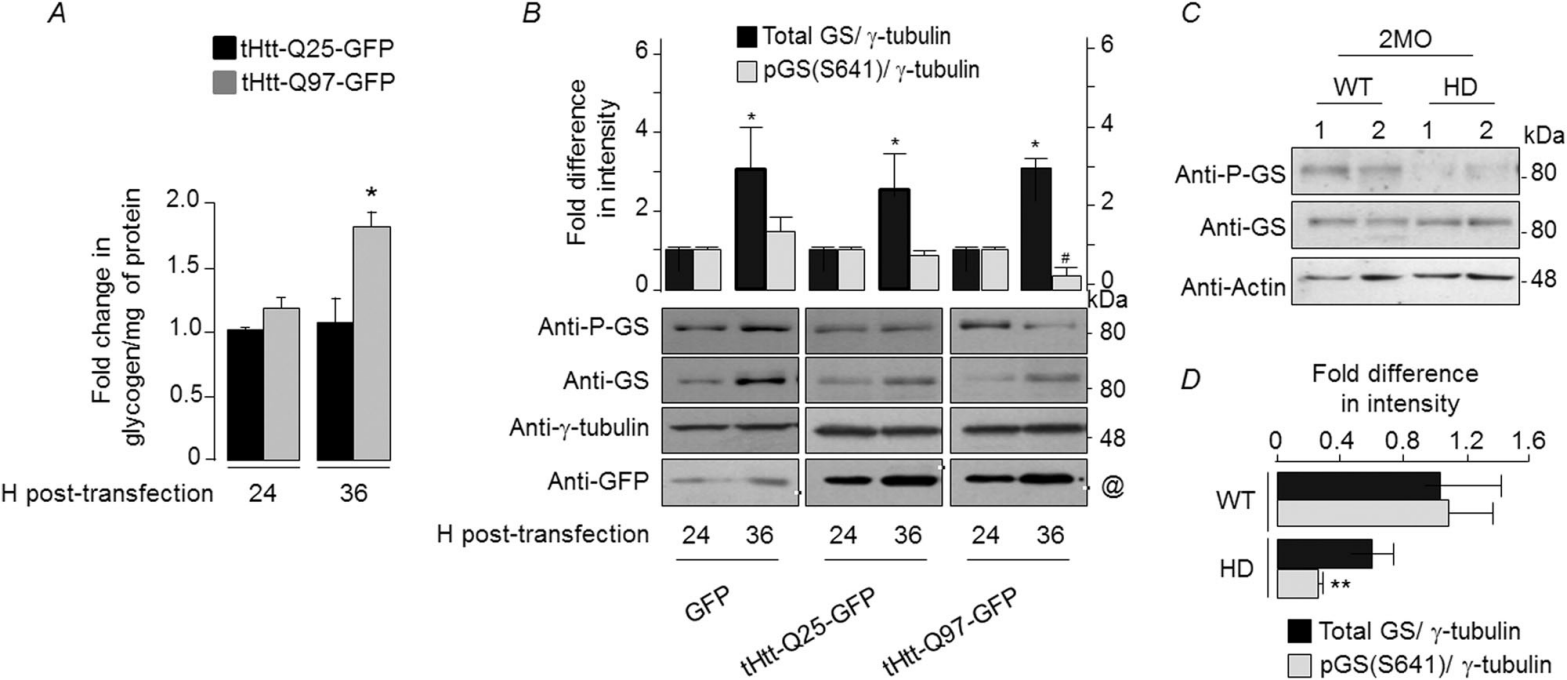
Activation of glycogen synthase could underlie the increased neuronal glycogen in Huntington disease models. **a** Bar diagram showing fold change in glycogen (normalized to proteins) in Neuro2A transiently expressing the tHtt-Q25-GFP or tHtt-Q97-GFP, harvested at 24 or 36 h post-transfection, as indicated. The glycogen level of the tHtt-Q25-GFP-expressing cells and harvested at 24 h post-transfection was considered as 1, and the relative levels in the other set was plotted (*N* = 3; *t*-test; *p ≤ 0.05). **b** Representative immunoblots showing the relative levels of total GS and its phospho-S641 form in Neuro2A cells transiently expressing the GFP, tHtt-Q25-GFP, or tHtt-Q97-GFP and harvested at 24 or 36 h post-transfection, as indicated. Probing for γ-tubulin served as loading control. Bar diagram above shows the fold changes in the signal intensities (measured by densiometric analysis) of the total GS and the phospho-S641 GS, both normalized to γ-tubulin level. The signal intensities obtained for the GS and the phospho-GS at 24 h post-transfection was considered as 1, the relative levels in the other set was plotted (*N* = 3; *t*-test; * or ^#^, *p* ≤ 0.05; *, compared with total GS level at 24 h; ^#^, compared with phospho-GS at 24 h). The molecular reference bands for the immunoblots probed for the GFP, tHtt-Q25-GFP, and tHtt-Q97-GFP expression are marked with a black/white line (~33, 42, and 80 kDa bands, respectively; identified with a “@” mark in the lower, “anti-GFP” panel). **c** Representative immunoblots showing the relative levels of total and phospho-S641 GS in the brain tissues from the 2-month-old (2MO) R6/2 mouse line (HD) and age-matched wild-type (WT) line (one pair each). Probing for actin served as the loading control. Note the reduction in the level of phospho-S641 GS in the HD brain as compared with the WT brain. **d** Bar diagram representing the fold changes in the signal intensities (measured by densiometric analysis) of the total GS and the phospho-S641 GS, normalized to β-actin levels, in the brain tissues from the R6/2 mouse line (HD) and age-matched WT line. The normalized signal intensities obtained for the GS and the phospho-GS in one of the WTs was considered as 1, the relative levels in the other set was plotted as fold change (N for WT = 4 and for HD = 3; *t*-test; **p ≤ 0.01; the fold change observed for the total GS was statistically not significant)

We next analyzed the activity of GS, the key enzyme involved in glycogen synthesis and whose activity is negatively regulated by phosphorylation at Ser641 residue^21^. Neuronal cells exposed to proteasomal or oxidative stress showed a significant reduction in the phospho-GS (Supplementary Fig. S4A,B) indicating that the stress-induced activation of GS could underlie the increased glycogen levels in such conditions (Supplementary Fig. S1A,B). To check for a similar response in HD, Neuro2A cells transiently expressing the mutant huntingtin were harvested at 24 or 36 h post-transfection, and the level of total and phospho-Ser641 form of GS was measured by immunoblotting. There was a significant difference in the glycogen level in the cells that overexpressed the tHtt-Q97-GFP and harvested at 36 h post-transfection (Fig. 2a) and this increase in glycogen level correlated with the decreased levels of the phospho-Ser641 GS as compared with cells that overexpressed GFP or tHtt-Q25-GFP (Fig. 2b). Intriguingly, the total GS level increased along the two time points in all three sets (Fig. 2b), and in the untransfected cells (Supplementary Fig. S4C), suggesting that the change observed for the total GS is independent of the GFP or the tHtt-GFP expression. However, the relative change in the phospho-Ser641 GS is likely to be a specific response to the expression of mutant huntingtin and stress. Indeed, a similar trend was also seen in the brain tissues of the R6/2 transgenic line (Figs. 2c, d).

### Increased GS activity protects Neuro2A cells from the mutant huntingtin-induced cytotoxicity

We next modulated the intracellular GS activity by overexpressing the GS or by its knockdown via RNA interference (RNAi), and measured the cytoxicity conferred by the huntingtin mutant protein. We have also used an expression construct coding for the glycogen-targeting subunit of protein phosphatase-1 (PTG/R5), which is known to activate GS and glycogen synthesis^7,22^. Overexpression of a construct coding for GS or PTG/R5 is known to increase intracellular glycogen level (see supplementary Fig. S5A,B)^7,22^ and thus are an indicator of increased GS activity. Overexpression of the mutant huntingtin in Neuro2A cells that were partially silenced for the GS led to a significant reduction in the glycogen level, as well as cell survival (Supplementary Fig. S6A). On the other hand, coexpression of GS or R5/PTG led to a significant reduction in the cytotoxicity induced by the mutant huntingtin (Supplementary Fig. S6B), suggesting a correlation between the intracellular glycogen level, GS activity and the cytotoxicity conferred by the mutant huntingtin.

**Fig. 3:**
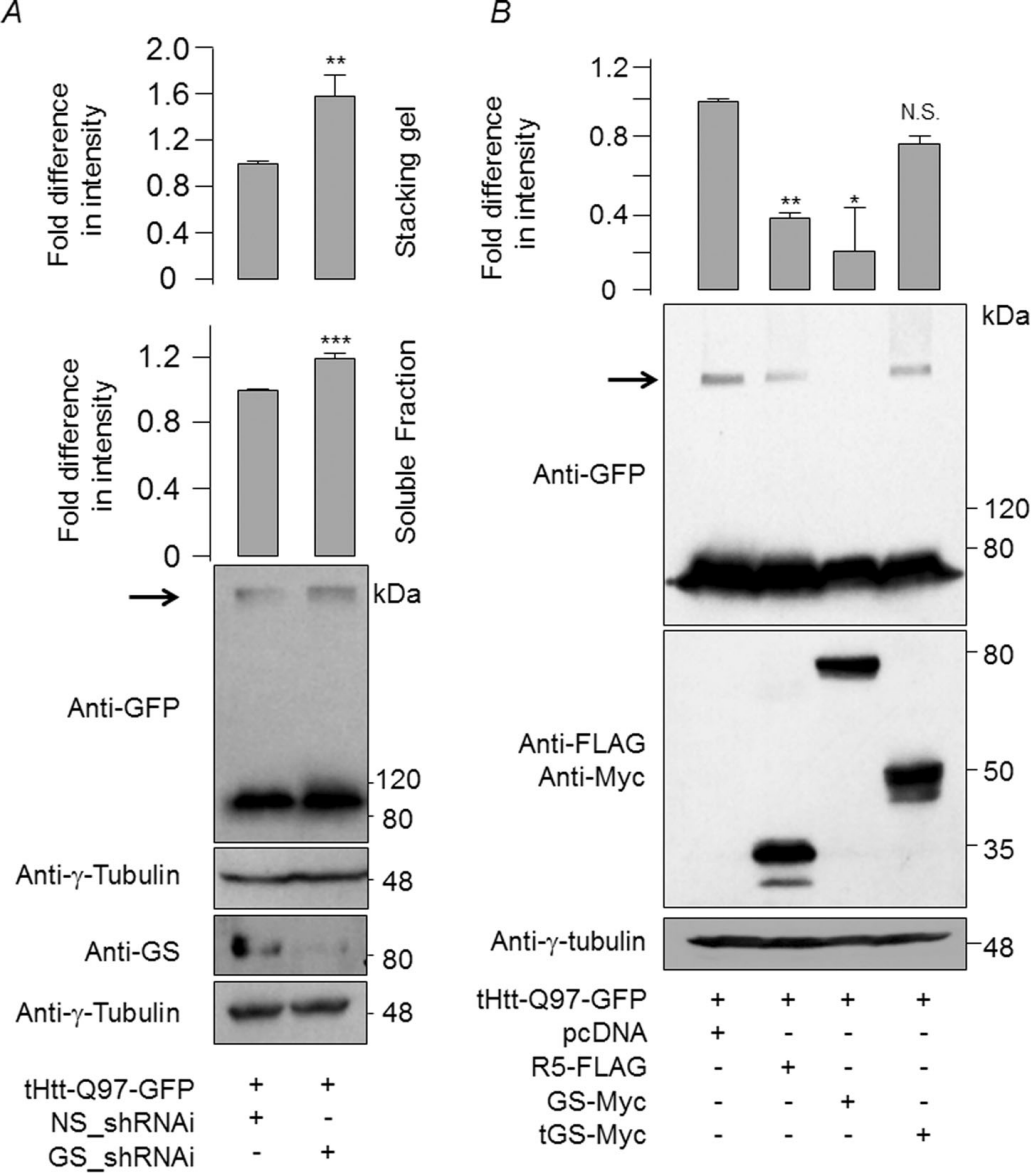
Glycogen synthase is required for the clearance of the high molecular weight, aggregated form of mutant huntingtin. **a** Immunoblot showing the relative levels of the soluble, and the insoluble high molecular aggregated forms (identified by an arrow) of mutant huntingtin from Neuro2A cells that transiently coexpressed the tHtt-Q97-GFP in the presence of a knockdown construct for GS (GS_shRNAi) or with a non-silencing control construct (NS_shRNAi). The bar diagram above shows the relative difference in the signal intensities of the mutant huntingtin in the soluble and insoluble fractions, as measured by a densiometric analysis of the blots. For this, the signal intensity of the band representing the cells transfected with the NS_shRNAi was considered as 1 (*N* = 3; *t*-test; **p ≤ 0.01; ***p ≤ 0.001). **b** Representative immunoblots showing the relative levels of insoluble, high molecular aggregated forms of mutant huntingtin trapped in the stacking gel (identified by an arrow) from Neuro2A cells that transiently coexpressed the tHtt-Q97-GFP with R5, GS, or the truncated GS (tGS), as indicated. The bar diagram above represents the fold change in the signal intensities (measured by densiometric analysis) of the huntingtin signal trapped at the stacking gel. Signal intensity in empty vector (pcDNA) was considered as 1 (*N* = 3; *t*-test; *p ≤ 0.05; **p ≤ 0.01; N.S., statistically not significant)

Next, we explored how GS might protect the cells from the cytotoxicity of mutant huntingtin. As shown in Fig. 3a, GS knock-down led to the increased levels of slow moving, aggregated form of the mutant huntingtin. Conversely, the overexpression of GS or PTG/R5, led to a significant reduction in the aggregated form of mutant huntingtin (Fig. 3b). Similarly, the overexpression of GS or PTG/R5 did reduce the level of mutant huntingtin in the insoluble fraction (Supplementary Fig. S7). We also counted the number of cells that showed mutant huntingtin, observed under a microscope. As shown in Supplementary Fig. S8A,B, a significant reduction in the number of aggregate-positive cells when the mutant huntingtin was coexpressed with the GS or PTG/R5; a majority of such cells showed diffused staining for the mutant huntingtin. We next wanted to test if overexpression of GS or R5 would also reduce the oxidative stress induced by the cytotoxic mutant huntingtin. As shown in Supplementary Fig. S9, the cellular level of SOD1 was much lower in the cells that were coexpressed with the mutant huntingtin and R5 or GS, suggesting a direct correlation between the insoluble form of huntingtin and the level of reactive oxygen species.

### GS protects neurons from physiological stress by increasing the autophagic flux

**Fig. 4:**
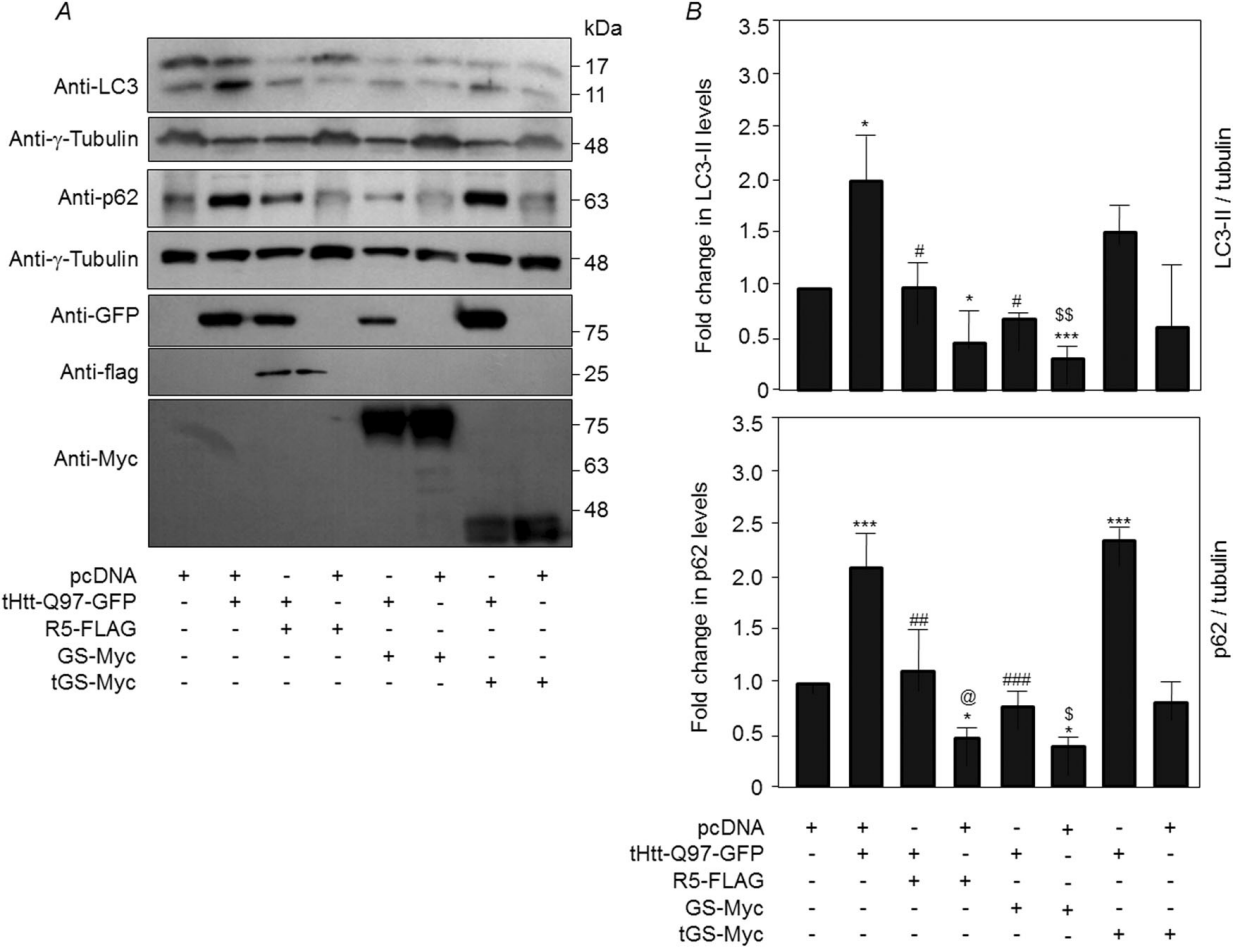
Overexpression of glycogen synthase reduces high molecular aggregates of mutant huntingtin by enhancing the autophagy flux. **a** Representative images of the immunoblots showing the relative levels of LC3 and p62 in Neuro2A cells transiently expressing R5, GS, or the truncated GS (tGS), either alone or along with tHttt-Q97-GFP, as indicated. Probing for tubulin served as the loading control, and probing for GFP or the FLAG/Myc epitope confirmed the expression of the constructs. **b** Bar diagram showing the fold changes in the signal intensities (measured by densiometric analysis) of the p62 or the LC3-II, both normalized to γ-tubulin level, from Neuro2A cells expressing the indicated combination of the constructs. The signal intensities obtained for the p62 and LC3-II bands for cell transfected with the empty vector (pcDNA) was considered as 1, their relative levels in the other sets were plotted (*N* = 3; *t-*test when two sets are compared, or one-way ANOVA Dunnett’s multiple comparison test when the control set was compared with two or more experimental sets; *, #, @ or $, *p* ≤ 0.05; ## or $$, *p* ≤ 0.01; *** or ###, *p* ≤ 0.001; * denotes the significance when the comparison was made with the cells expressing pcDNA; # with cells coexpressing tHtt-Q97-GFP and pcDNA; @ with cells coexpressing tHtt-Q97-GFP and R5-FLAG; $ with cells coexpressing tHtt-Q97-GFP and GS-Myc)

**Fig. 5:**
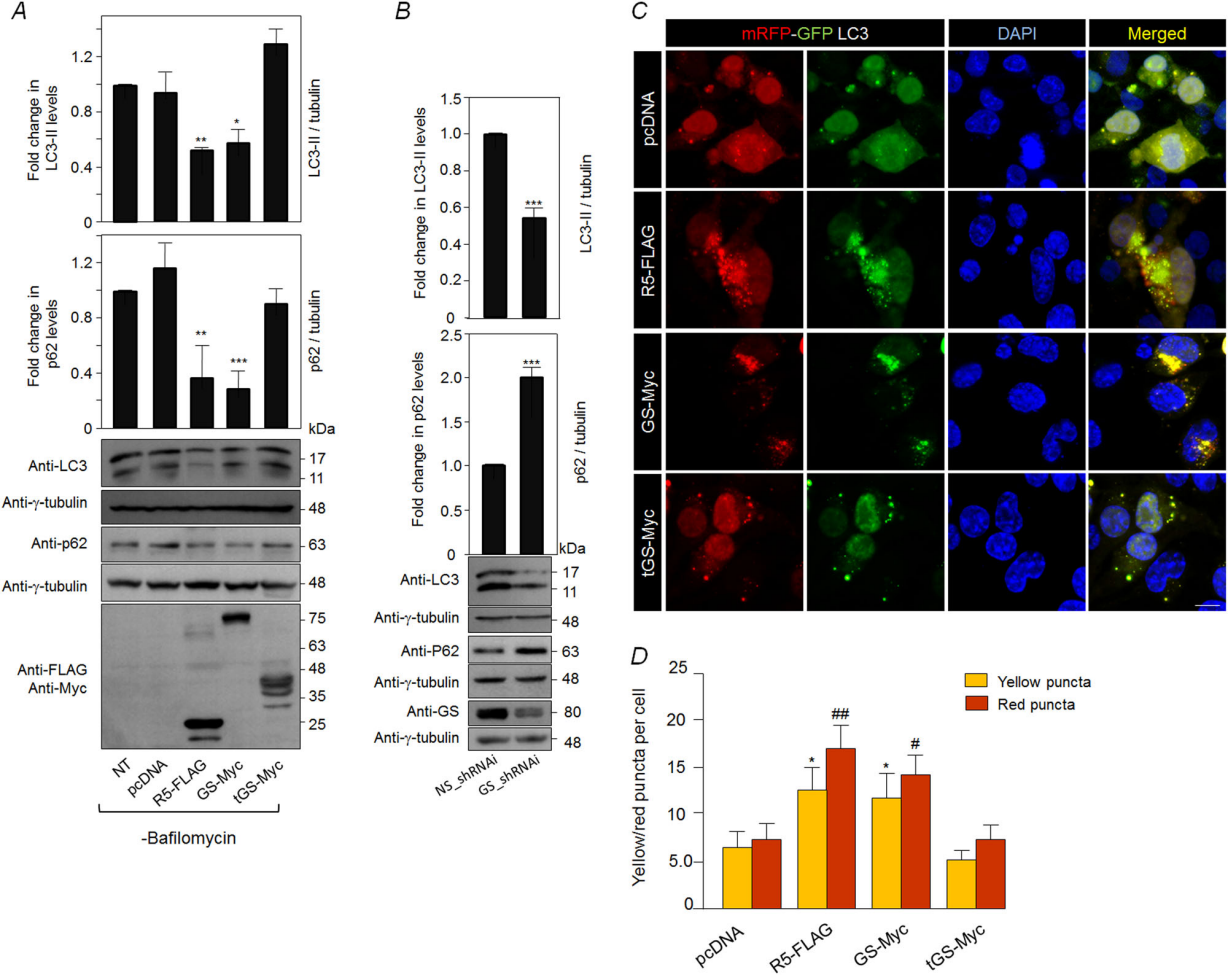
Glycogen synthase regulates autophagy flux in Neuro2A cells. **a** Representative immunoblots showing the relative levels of LC3 and p62 in Neuro2A cells transiently overexpressing R5, GS, the truncated GS (tGS), or the empty vector (pcDNA) either in the absence (−) (Fig. 5a) or presence (+) (see Supplementary Fig S11A-B) of bafilomycin (an autophagic blocker), as indicated. Probing for γ-tubulin served as the loading control, and probing for FLAG/Myc epitope confirmed the expression of the constructs. Bar diagram, above, shows the fold changes in the signal intensities (measured by densiometric analysis) of the p62 or the LC3-II, both normalized to γ-tubulin level. The signal intensities obtained for p62 and LC3-II for the control set (non-transfected (NT) cells) was considered as 1, the relative levels in the other sets were plotted (*N* = 3; *t*-test; *p ≤ 0.05; **p ≤ 0.01; ***p ≤ 0.001). **b** Representative immunoblots showing the relative levels of LC3 and p62 in Neuro2A cells transiently transfected with the knockdown construct for GS (GS_shRNAi). A non-silencing control construct (NS_shRNAi) was used to normalize the plasmid DNA in the transfection. Probing for γ-tubulin served as the loading control. Bar diagram, shown above, represent the fold changes in the signal intensities (measured by densiometric analysis) of the p62 or the LC3-II, both normalized to γ-tubulin level. The signal intensities obtained for the set transfected only with the non-silencing construct was considered as 1, and the relative levels in the other set was plotted (*N* = 3; *t-*test; ***p ≤ 0.001). **c** Representative fluorescence images showing the red/green signals from the mRFP-GFP-LC3 construct for measuring the autophagic flux when expressed alone (pcDNA) or in combination with R5, GS, or the truncated GS (tGS), as indicated. Nuclei were stained with DAPI (blue). Note the increased number of red/green puncta in cells expressing R5 or GS. Scale bar, 10 µm. **d** Bar diagram showing average number of yellow and red LC3-positive puncta in Neuro2A cells coexpressing empty vector (pcDNA), R5, GS, or the truncated (tGS), as indicated (*N* = 3; *t*-test; * or #, *p* ≤ 0.05; ##, *p* ≤ 0.01; * compared with yellow puncta in cells transiently coexpressed with pcDNA and mRFP-GFP-LC3, and # compared with red puncta in cells coexpressing pcDNA and mRFP-GFP-LC3)

Given the observations mentioned above, we reasoned that the Neuro2A cells coexpressing tHtt-Q97-GFP with R5 or GS might clear the toxic aggregates by enhancing the autophagy flux and tested the same by measuring the levels of p62 and LC3-II—the two established readouts of autophagy flux^23,24^. As shown in Figs. 4a, b, coexpression of tHtt-Q97-GFP along with GS or R5 restored the autophagy flux to an extent comparable to the control set (pcDNA-transfected cells). As excessive autophagy is known to induce neuronal death^25–27^ (Supplementary Fig. S10), we reasoned that activation of GS in unstressed neuronal cells might induce autophagy and the autophagy-mediated cell death. As shown in Fig. 5a, overexpression of GS or R5 led to a significant reduction in the cellular levels of LC3-II and p62, suggesting that the increased GS activity level could lead to increased autophagy flux. This change in level, however, was restored upon addition of bafilomycin A1, an autophagy blocker, (Supplementary Fig. S11A,B), suggests that the observed changes are indeed due to the enhanced autophagic flux. Conversely, the RNAi-mediated depletion of GS lead to significant increase in the level of p62 and a significant reduction in the level of LC3-II band (Fig. 5b), suggesting compromised autophagy and a regulatory role for GS in autophagy induction. To further strengthen our observations, we used the tandem mRFP-GFP-LC3 construct coding for a pH-sensitive fusion protein, a well-established tool for measuring autophagy flux^28^. The green-red/yellow puncta indicate autophagosomes, whereas the autolysosome (autophagosome fused to lysosome) would be red in color. As shown in Figs. 5c, d, the coexpression of GS or R5 led to a significant increase in the number of cytoplasmic LC3-positive puncta that were positive only for the red and the red-green (yellow) double-positive puncta, further confirming the notion that GS induces autophagy.

**Fig. 6:**
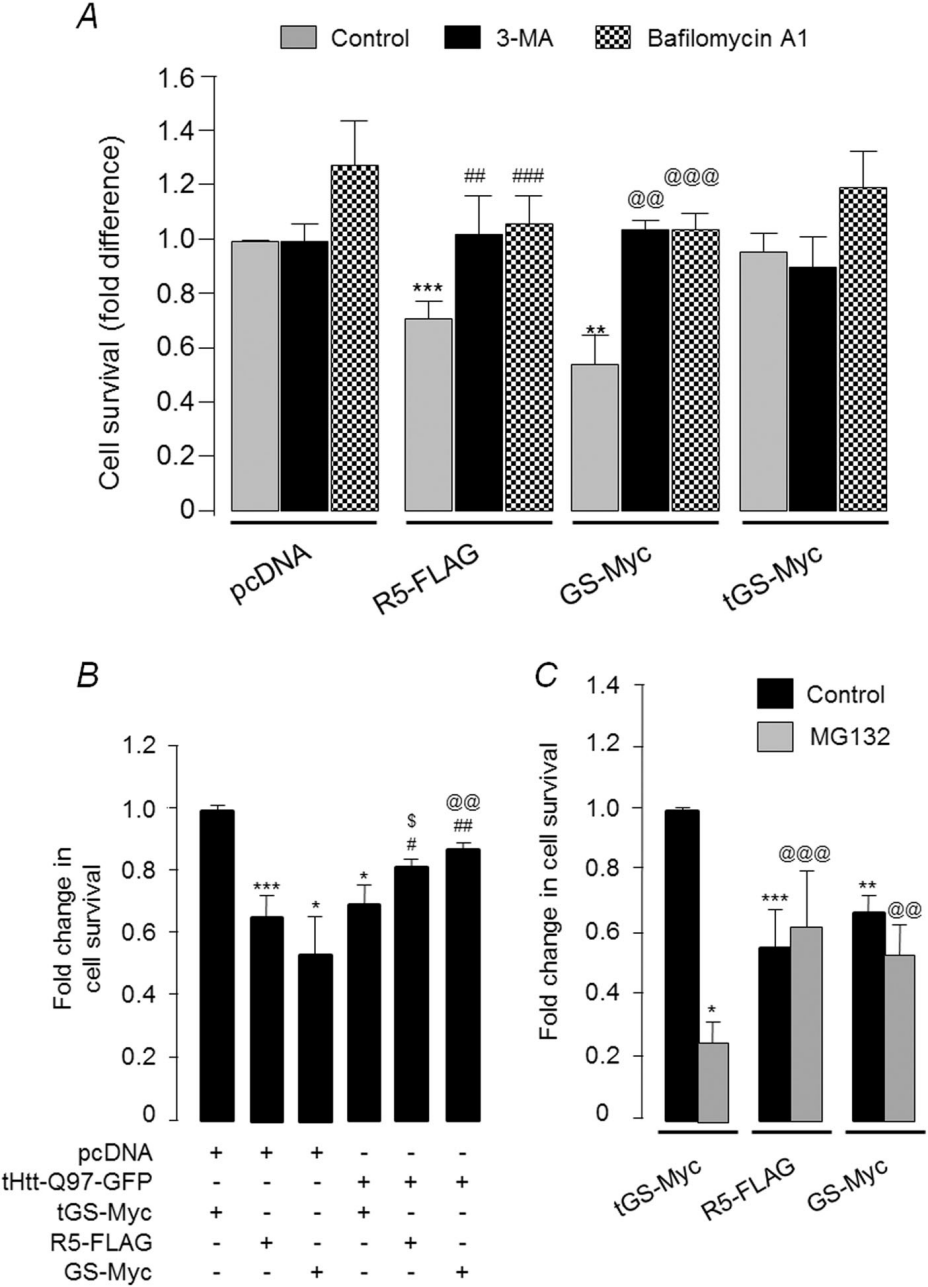
Glycogen synthase-induced autophagy induction is cytotoxic to unstressed Neuro2A cells. **a** Bar diagram showing the fold change in survival of Neuro2A cells transiently expressing R5, GS, or the truncated GS (tGS) in the presence or absence of autophagic blockers 3-MA and Bafilomycin A1. Cells treated with the vehicle (DMSO) served as the control. The survival rate of Neuro2A cells transfected with the empty vector (pcDNA) and vehicle treated was considered as 1, the relative survival of cells in the other sets were plotted (*N* = 3; *t*-test when only two sets are compared and one-way ANOVA Dunnett’s multiple comparison test when one control set was compared with two or more experimental sets; **, @@ or ##, *p* ≤ 0.01; ***, @@@ or ###, *p* ≤ 0.001; * denotes significance when compared with pcDNA expressing cells; # when compared with R5-FLAG expressing cells;@ when compared with GS-Myc expressing cells). **b** Bar diagram showing fold change in the survival of Neuro2A cells when R5 or GS were transiently expressed either alone or along with tHtt-Q97-GFP, as indicated. The survival rate, as measured by MTT assay, of the cells that expressed the truncated GS (tGS) was considered as 1, the fold difference in the survival for other sets was plotted (*N* = 3; t-test when only two sets are compared and one-way ANOVA Dunnett’s multiple comparison test when one control set is compared with two or more experimental sets; *,# or $, *p* ≤ 0.05; ## or @@, *p* ≤ 0.01; ***p ≤ 0.001; * was used to denote significance when compared with cells coexpressing pcDNA and tGS-Myc; # when compared with cells expressing tGS-Myc along with tHTT-Q97-GFP; $ when compared with cells coexpressing R5-FLAG and pcDNA; @ when compared with cells coexpressing GS-Myc and pcDNA). **c** Bar diagram showing fold change in survival of Neuro2A cells transiently expressing R5, GS or the truncated GS (tGS) and treated with either the vehicle DMSO (control) or a proteasomal blocker (MG132 40 µM) for 6 h. The cell survival was measured by MTT assay. Values were normalized to cells expressing the tGS (*N* = 3; *t*-test when only two sets were compared and one-way ANOVA Dunnett’s multiple comparison test when one control set was compared with two or more experimental sets; *p ≤ 0.05; ** or @@, *p* ≤ 0.01; *** or @@@, *p* ≤ 0.001; * is used to denote significance when compared with cells expressing tGS-Myc (vehicle); @ is used when cells expressing tGS-Myc and treated with MG132 is used for comparison)

Having established a role for GS in autophagy flux, we next tested whether the autophagy induction is indeed the cause of the overexpressed GS-mediated death in the Neuro2A cells under “non-stress” conditions. As shown in Fig. 6a, the 3-methyladenine (3-MA) or Bafilomycin (both are autophagy blockers) addition to the medium led to a significant increase in the survival of cells overexpressing the GS or R5, and the survival was comparable to the cells that expressed the empty vector or the truncated GS. Taken together, the results suggest that excessive autophagy could be the cause of the death of Neuro2A cells overexpressing GS or R5.

As the increased GS activity appears to protect the Neuro2A cells against the cytotoxicity of mutant huntingtin (Fig. 6b), we next wanted to check that whether increased activity of GS could protect Neuro2A cells against other forms of physiological stress. To test this, Neuro2A cells overexpressing GS or R5 were partially blocked for proteasomal activity and the cell viability was measured. As shown in Fig. 6c, overexpression of GS or R5 could rescue the MG132-mediated cytotoxicity, suggesting that the increased GS level/activity is cytotoxic only when the neuronal cells are not under physiological stress. However, overexpression of R5 or GS in a non-neuronal cell line (COS-7) was not cytotoxic (Supplementary Fig. S11C), suggesting that the toxicity observed for the overexpressed GS was specific to neuronal cells.

### Cellular glycogen might modulate the aggregation kinetics of polyglutamine protein

**Fig. 7:**
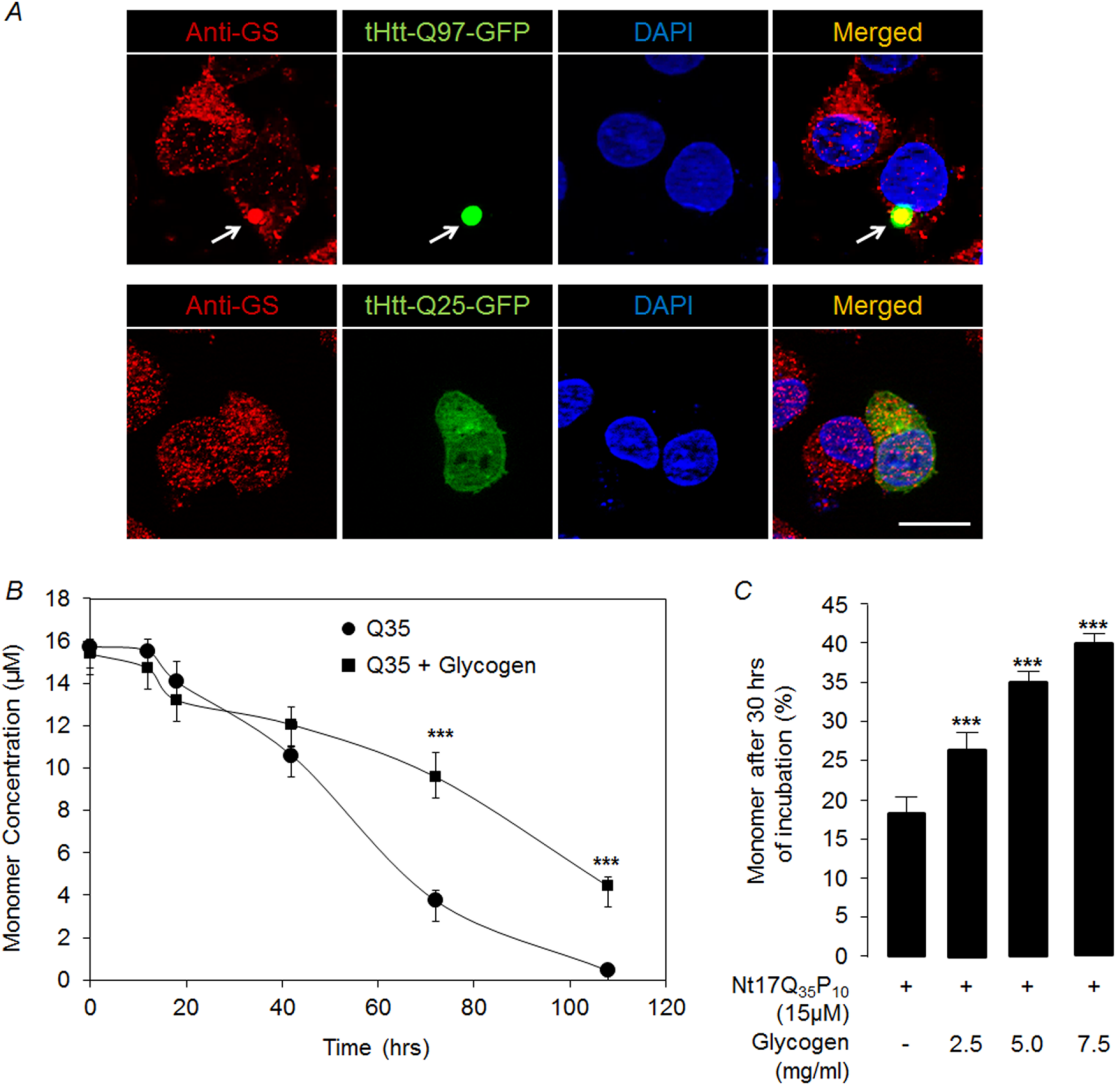
Pure glycogen delays aggregation kinetics of polyglutamine peptide in vitro. **a** Representative fluorescence images of Neuro2A cells transiently expressing tHtt-Q25-GFP or tHtt-Q97-GFP immunostained with an antibody to detect the endogenous glycogen synthase (red). Nuclei were stained with DAPI (blue). Note the GS localization with GFP-positive aggregates of mutant huntingtin (arrow). Scale bar, 10 µm. **b** A line diagram showing the levels of the monomeric form of the synthetic polyglutamine peptide (Q35) (in µM), as determined by RP-HPLC, when incubated alone or with pure glycogen (5 mg/ml) at indicated time points (*N* = 3; one-way ANOVA with Holm–Sidak post-hoc testing; ***p ≤ 0.001). (**c**) Bar diagram showing percent monomers detected after 30 h of incubation of Nt_17_Q_35_P_10_ in the presence of increasing concentration of pure glycogen (0, 2.5, 5, 7.5 mg/ml) (*N* = 3; one-way ANOVA with Holm–Sidak post-hoc testing; ***p ≤ 0.001)

As the stress-induced activation of GS results in increased glycogen levels, we next wanted to explore the possible role of glycogen in preventing/clearing the abnormally aggregated protein. We found that GS colocalize with the mutant huntingtin aggregates (Fig. 7a) and also the glycogen particles localize on or around the aggregates of the mutant huntingtin in the Neuro2A cells (Supplementary Fig S2). To test if the glycogen can modulate the aggregation properties of the polyglutamine proteins, we used an established in vitro assay to look at the aggregation kinetics of peptide bearing glutamine repeats^29–33^. We found that presence of pure glycogen delayed the aggregation kinetics of the polyglutamine peptide and this effect was dose dependent (Figs. 7b, c). Thus, glycogen is likely to reduce the aggregation of the mutant huntingtin with expanded polyglutamine repeats in the cellular environment as well. To test this possibility, we partially silenced the GS in Neuro2A cells, induced autophagy using rapamycin, and looked at the levels of aggregated forms of the huntingtin. As shown in Supplementary Fig.S12A,B, rapamycin treatment could clear the huntingtin aggregates in the cells partially silenced for GS. Thus, the possible role of glycogen in the aggregation/clearance of mutant huntingtin in the cellular environment needs to be explored further.

### Neuronal glycogen inclusions are common in neurodegenerative disorders

**Fig. 8:**
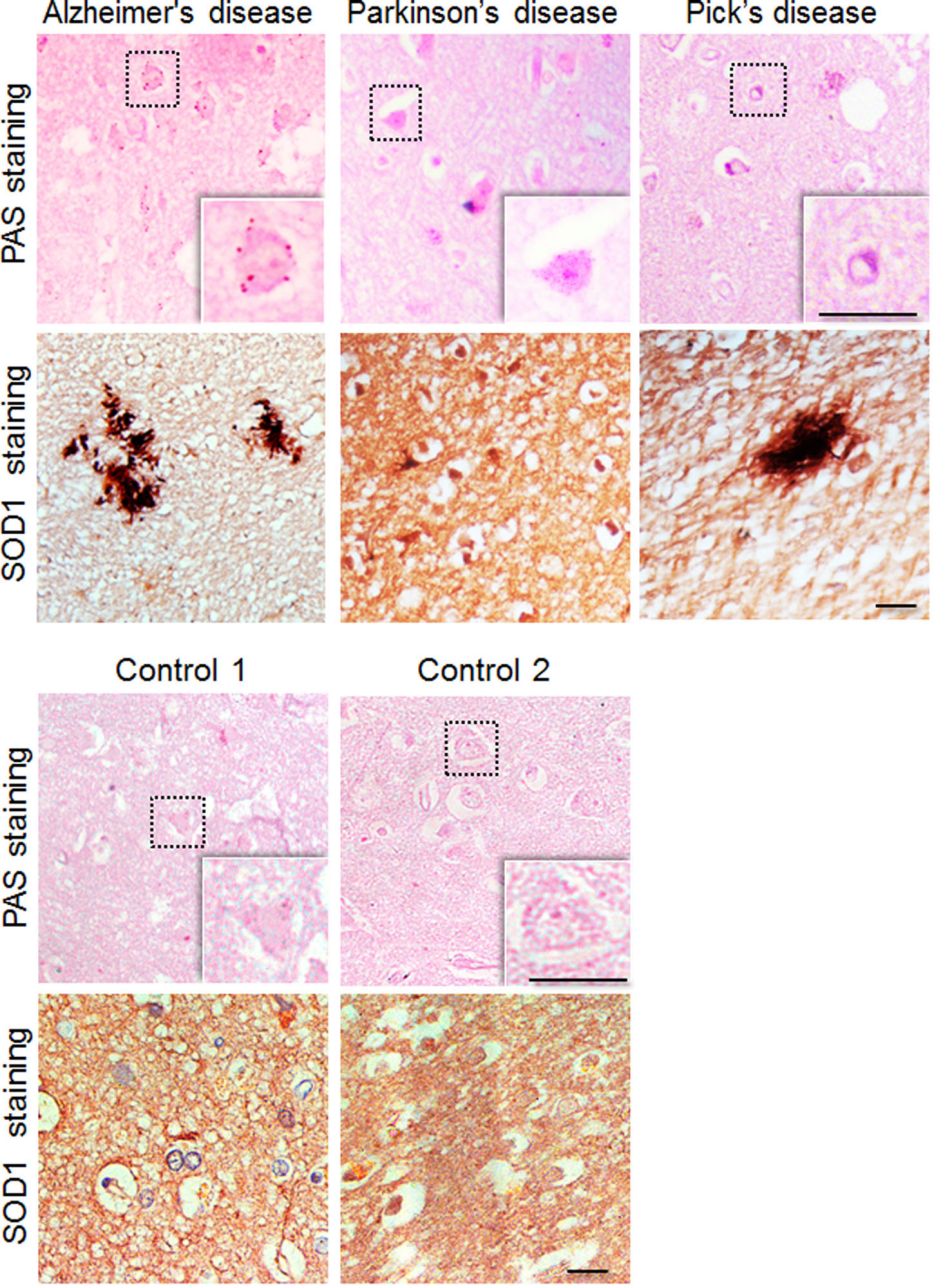
Glycogen inclusions in the frontal cortex region of subjects with neurodegenerative disorders. Representative bright field images showing PAS-positive glycogen granules in the frontal cortex region of autopsied brain tissues of subjects clinically diagnosed to have Alzheimer’s disease, Pick’s disease, or Parkinson’s disease as identified. The same regions of the brain were also immunostained with the SOD1 antibody, revealing increased levels of immunoreactivity. The panel below shows PAS and SOD1 immunoreactivity of the frontal cortex area of the two age-matched controls. These sections were from autopsied brain tissues of subjects who did not suffer from neurodegenerative conditions. Details on the subjects are given in the Supplementary Table S1. The PAS-positive glycogen granules shown here did not show autofluorescence, indicating they are not lipofuscin granules (scale bar, 10 µm)

As physiological stress underlies neurodegeneration, we wanted to test if glycogen accumulation could be seen in other neurodegenerative disorders. For this, tissue sections from the frontal cortex region of autopsied brain samples of individuals clinically diagnosed to have Alzheimer’s disease, Pick’s disease, or the Parkinson’s disease and from age-matched normal (control) individuals were used (see Supplementary Table S1 for details of subjects)^34^. As shown in Fig. 8, PAS staining revealed numerous neurons with PAS-positive granules, confirming the glycogen accumulation, in the three neurodegenerative conditions tested, compared with the two age-matched control brains (Fig. 8).The brain sections of the all three affected showed SOD1-positive inclusions, although to varying dimensions, whereas the age-matched control brain showed a diffused cytoplasmic staining for the SOD1, suggesting physiological stress in affected individuals.

## Discussion

While a few studies have shown a positive correlation between glycogen levels and neurodegeneration^6,7^, the significance of glycogen and the glycogen synthetic machinery in the neuronal physiology has not been elucidated. In this study, we show that the expression of the mutant huntingtin-induced neuronal glycogen synthesis, and that the activation of GS could inhibit the aggregation of the mutant protein. Our findings provide a novel insight into the neuronal functions of glycogen synthetic machinery that are beyond the traditionally recognized cellular roles for this process.

One of the intriguing observations of our present study is the observed link between the GS and autophagy in Neuro2A cells. The GS-dependent autophagy flux could underlie neuronal protection against the cytotoxic mutant huntingtin. Intriguingly, however, the GS-associated autophagy induction appears to protect the neuronal cells only under physiological stress. Forced expression of GS or PTG reduced the survival of Neuro2A cells. However, autophagy blockade restored survival of cells overexpressing GS or R5, suggesting that excessive autophagy is detrimental when neuronal cells are not under stress. Indeed excessive autophagy is shown to cause cell death in cortical neurons^35,36^, and in cerebral ischemia^27,37^ and similar observations were made, by us (present study) and others^38^. The forced expression of GS in neuronal cells through transgenesis in *Drosophila* and mouse lead to excessive neuronal death and decreased lifespan^8^, possibly due to the increased autophagy flux. Emerging reports strengthen our conclusions that the end effect of autophagy flux in neuronal cells could highly be contextual and thus autophagy can either promote the survival or the death of the neurons depending on the neurophysiological states^38^. However, whether the autophagy flux could also be a consequence of the glycogen build-up is yet to be resolved. As glycogen is known to be degraded via autolysosomes on very high energy demand^39^, and as such metabolic processes have feedback loops^40^ and that the metabolites of carbohydrate are also known to induce autophagy^41^, it could be argued that the increased glycogen could also lead to increased autophagic flux in neuronal cells. Indeed GS is known to regulate the autophagic process, by interacting with Atg8 in *Drosophila*^42^, but whether similar levels of activation of autophagy can be achieved in absence of glycogen is yet to be deciphered. Similarly the AMP-activated protein kinase, a key energy sensor in the cell and a key regulator of GS^43,44^, is known to regulate the level of glycogen via the autophagic process under nutrient deficient conditions^45^. Conversely, accumulation of abnormal glycogen is known to associate with autophagy blockade and neurodegeneration in the mouse models of Lafora disease^46–48^. Clearly, further work is required to understand the functional link between glycogen, GS, and autophagy induction in neuronal cells.

We have shown earlier that glycogen particles are recruited to aggresomal structures through an active process upon proteasomal blockade^49^, suggesting a causal role for glycogen in the proteolytic processes. Extending this suggestion, we show here that glycogen particles are recruited to the aggregates of mutant huntingtin in Neuro2A cells, and that an increase in the glycogen level—a direct fall out of increased GS activity—inversely correlates with the aggregation of the mutant huntingtin, its insolubility, and cytotoxicity. Thus, the stress-activated GS could have two distinct roles; one to induce autophagy and the other to increase neuronal glycogen as a stress response mechanism. For example, the glycogen synthesized under stress might lower the toxicity of mutant huntingtin by preventing its aggregation as pure glycogen delayed the aggregation of polyglutamine peptides in vitro. We propose that glycogen might delay the aggregation by two distinct mechanisms; due to its macromolecular crowding effect within the cell, and by meeting the energy demand of the proteolytic center. Indeed macromolecular crowding agents are known to inhibit the aggregation of β-rich protein, like bovine carbonic dehydrogenase^50^, and polyglutamine peptide have been shown to form aggregates in vitro, which are rich in β-sheet like structure^51^. Also, glycogen is known to serve as a crowding agent^52^. It is known that the misfolded proteins are recruited to aggresomes^53,54^ and that mitochondria and glycogen particles cluster around the aggresome, possibly to provide energy to the proteolytic processes^49,53^. Thus, the increased glycogen and its colocalization with the huntingtin aggregates might help in facilitating their clearance via the proteolytic processes. A third and an equally compelling possibility could be that glycogen function as a scaffold agent thereby facilitating the proximity of proteolytic machinery with the protein aggregates. Indeed a scaffolding function was known for glycogen^55^ and it would indeed be interesting to test such a possibility in the aggresomal functions.

Notwithstanding the initial trigger, oxidative stress appears to be a common underlying theme in a diverse set of neurodegenerative disorders, more likely due to the very high demand for oxygen and the high rate of metabolism in the neurons^56^. Extending our observations in the Neuro2A cells, we have also found glycogen accumulation in the post-mortem brain sections of patients with Alzheimer’s disease, Pick’s disease, and Parkinson’s disease, and show that the oxidative stress, as measured for SOD1 immunoreactivity correlated with the glycogen accumulation. Thus, glycogen in these brain sections possibly represents a failed attempt by the neurons to cope-up with the neuronal stress. This may perhaps explain why glycogen granules are seen only in the neurons of the aged brain and in neurodegenerative disorders. Therefore, it might be of interest to test if forced and regulated induction of glycogen synthesis can prevent neurodegeneration in the HD mice models.

## Materials and methods

### Expression constructs and reagents used

The expression constructs coding for the tHtt-Q97-GFP and tHtt-Q25-GFP were a generous gift from Professor Lawrence Marsh (University of California at Irvine, USA), and the expression construct coding for the PTG/R5 was a generous gift of Professor Alan R. Saltiel (University of California, USA). The expression constructs coding for the full-length and the truncated versions of the GS are reported in our previous studies^57^. The construct coding for the tandem fluorescent-tagged LC3 (mRFP-GFP-LC3) was a generous gift from Dr Tamotsu Yoshimori, (National Institute for Basic Biology, Japan). Amyloglucosidase, Bafilomycin, 3-MA, and MG132 were purchased from Sigma-Aldrich India Pvt. Ltd; rapamycin was purchased from Enzo Life Sciences (USA); peptide bearing polyglutamine repeats (Q35 peptide) was synthesized at Keck Biotechnology Resource Laboratory, Yale University, USA.

### Antibodies

The following antibodies were used in this study: anti-GFP (11814460001; IB, 1:3000) and anti-Myc (11667149001; IB, 1:3000) from Roche Products India Pvt. Ltd.; anti-FLAG (F7425; IB, 1:2000) and anti-γ-tubulin (T6557; IB, 1:10,000) were from Sigma-Aldrich India Pvt. Ltd; anti-GS (3893; IB, 1:1000, IC: 1:50), anti-phospho-GS (3891; IB, 1:1000), and anti-LC3B (2775; IB: 1:1000) were from Cell Signaling Technology, Inc. (USA); anti-p62/SQSTM1(BML-PW-9860; IB:1:1000) was from Enzo Life Sciences (USA); anti-SOD1 (1/407, IB: 1:1000; IHC 1:100) and anti-Htt (sc-8767; IHC 1:500) were from Santa Cruz Biotechnology (USA). Anti-glycogen antibody was discussed in earlier reports^15^ and was a generous gift from Dr Otto Baba (School of Dentistry, Ohu University, Japan). All secondary antibodies were procured from Jackson ImmunoResearch Inc. (USA) and DAB kit (1610500011730) was from Bangalore Genei Private Limited (India).

### Cell culture

The Neuro2A and COS-7 cell lines were obtained from the National Centre for Cell Science, Pune, and the HT-22 cell line was obtained from the National Brain Research Centre, Manesar. The neuroblastoma cell line Neuro2A was used for all experiments unless otherwise stated. Cells were grown at 37 °C, 5% CO_2_ in a humid environment. Complete Dulbecco’s modified Eagle's medium (Sigma-Aldrich India Pvt. Ltd) supplemented with 10% fetal bovine serum (Invitrogen, USA) and antibiotic cocktail (Sigma-Aldrich India Pvt. Ltd) was used to maintain the cell lines. Cells were transfected using Turbofect transfection reagent (Thermo Fisher Scientific) at 60–70% confluency. Cells around 80–90% confluency were passaged to maintain the running culture. DAPI staining was routinely performed to rule out mycoplasma contamination.

### Glycogen estimation

The cellular glycogen content was measured essentially as described earlier^19,40^ by measuring the glucose release upon digesting glycogen with amyloglucosidase (Sigma-Aldrich India Pvt. Ltd). Briefly, cells at 60–70% confluence were transfected using TurboFect Transfection Reagent (Thermo Fisher Scientific), and were harvested at 36 h post transfection. For glycogen estimation, cells were lysed in 100 µl of 30% KOH, and were boiled for 20 min at 100 °C. A small aliquot (20 µl) was used for protein estimation using the BCA method, and remaining sample (80 µl) was spotted on a filter paper (2 cm × 2 cm) (Whatman Filter Paper #31-ET CHR). The filter paper with the spotted lysate was washed in 66 % ethanol and dried overnight. Amyloglucosidase (1 mg/ml in 0.2 M sodium acetate buffer pH 4.8) was used to release glucose from glycogen spotted on the chromatography paper, and the released glucose was measured using a colorimetric assay kit (ERBA Diagnostics Mannheim Gmbh Ltd). The value obtained for the glucose level was normalized using the protein data, and fold change in the glycogen level was plotted. Each measurement was done in duplicate and a minimum of three experimental sets were used to arrive at the average value, standard deviation and statistical significance.

### Immunofluorescence staining

Immunofluorescence staining was carried out essentially as described earlier^13,58,59^. Briefly, cells at 36 h post-transfection were fixed using 4% paraformaldehyde in 1X phosphate-buffered saline (PBS) for 20 min. The fixed cells were permeabilized using 0.05% Triton X-100 and blocked using 5% equine serum and 5% fish gelatin for 45 min. Cells were subsequently incubated with the primary and secondary antibody according to the manufacturer’s protocol. To visualize the nuclei, the cells were counter stained with DAPI for 5 min. Fluorescence images were captured using the Axiovision Epifluorescence microscope fitted with the ApoTome module (Carl Zeiss, Bangalore, India) and the images were assembled using Adobe Photoshop. For quantifying the LC3-positive cytoplasmic puncta (red-green signal overlap), the captured images from three independent sets, and in each set two biological repeats were processed (approximately 30 cells in each set) using the colocalization macro in ImageJ software, as described earlier^13^. The Huntingtin aggregates were counted manually by a blind observer from three independent sets (approximately 100 cells in each set) of experiments.

### MTT assay

Equal number of cells was seeded in a 24-well plate for the cell survival assay as described^13,58^. Cells were transfected at 60–70% confluency and at 40 h post-transfection, the cells were incubated with 0.5 mg/ml of thiazolyl blue tetrazolium bromide (MTT) (Sigma-Aldrich India Pvt. Ltd) and chased for 2 h. The cells were incubated in dimethylsulfoxide (DMSO) for 20 min to dissolve the formazan crystals and the change in optical density was measured at 565 nm using SpectraMax M3 Multi-Mode Microplate Reader (Molecular Devices).

### Soluble–insoluble fractionation

For the fractionation of insoluble aggregated form of huntingtin with expanded polyglutamine repeat, the Neuro2A cells were lysed and the insoluble material was pelleted by centrifugation as described earlier^60^.

### Immunoblotting

Protein samples were prepared from cells harvested at 36 h post-transfection. Cells were lysed using the Laemmli buffer and the protein was estimated using BCA method. Equal amount of protein was resolved on a sodium dodecyl sulfate-polyacrylamide gel electrophoresis along with pre-stained molecular weight marker (range 11–180 kDa; Genetix Biotech Asia Pvt. Ltd, cat. no. P006–0500), transferred to nitrocellulose membrane and processed for immunoreactivity as described earlier^13,19^. The immunoreactive bands were detected with a chemiluminescent detection kit (Supersignal West Pico, Pierce). Signal intensities on the immunoblots were quantified using NIH image software (ImageJ) as described earlier^59^. Signal intensity for γ-tubulin or β-actin served as the loading control for the normalization.

### Immunohistochemistry and PAS staining

Immunohistochemical staining was done on paraformaldehyde-fixed, paraffin-embedded sections essentially as described previously^47^. For the PAS staining, the cells were fixed in Carnoy’s fixative (60% ethanol, 30% chloroform and 10% glacial acetic acid) for 1 h at −20 °C, washed subsequently with 66% ethanol and 100% ethanol, and processed for the staining. For the tissue, the deparaffinized sections were used for the staining using conventional histochemical procedure^61^. Brain sections from three wild-type and three transgenic mice were used for the PAS staining and images were captured using the Nikon ECLIPSE 80i microscope.

### Animals

The HD transgenic mouse line (R6/2), bearing the Htt exon 1 with ∼150 CAG repeats (strain name: B6CBA-Tg(HDexon1)62Gpb/3J) and obtained from the Jackson Laboratory, reported in an earlier study^62^, was used. The animals were genotyped as reported^62^. The study protocol was approved by the Institutional Animal Ethics Committee of National Brain Research Centre, Manesar. To isolate the frontal cortices, mouse brain was first dissected into the left and right hemisphere. The olfactory bulb was then removed and then a cut at the anterior tip (or genu) of the corpus callosum was made to isolate the brain region having the frontal cortex.

### Human brain samples

The archival brain tissues of Asian Indians collected at autopsy and stored at the National Human Brain Tissue Repository, NIMHANS, Bengalore^63^ was used for the study. The clinical diagnosis was of the subjects was made by trained neurologists. The age-matched control samples represent subjects who were neurologically normal but succumbed to road traffic accidents. The study was approved by the Institutional Human Ethics Committee of the NIMHANS. The age, gender, and post-mortem interval of the subject used are given in Supplementary Table S1.

### Polyglutamine aggregation assay

Commercially synthesized glutamine homopolymeric (Q35) peptide was purified, lyophilized, and disaggregated as per established protocol^32,64^. Aggregation reaction was carried out at a concentration of ~15 µM Q35/Nt17Q35P10 peptide and 2.5–7.5 mg/ml glycogen in PBS pH 7 with 0.05% sodium azide as an anti-microbial agent and was incubated at 37 °C. The aggregation reaction was monitored by taking small aliquots of the sample at different time points and analyzing using reverse phase high-performance liquid chromatography (RP-HPLC), as described earlier^64^.

### Statistical analysis

In the figures, each bar represents mean values of minimum three biological replicates and the error bar represents the standard deviation of the mean. Statistical significance was analyzed with two-tailed unpaired *t-*test using the GraphPad software or one-way analysis of variance using the Prism or the Sigma Plot software.

## Electronic supplementary material

Supplementary Figures
