# Supplementary material for: Glycogen synthase protects neurons from cytotoxicity of mutant huntingtin by enhancing the autophagy flux
Source: Cell Death Dis. 2018 Feb 8;9(2):201. doi: 10.1038/s41419-017-0190-5 (PMC5833817; doi:10.1038/s41419-017-0190-5)
Supplement: Supplementary file 1 — Supplementary Figures [file 41419_2017_190_MOESM1_ESM.pdf]

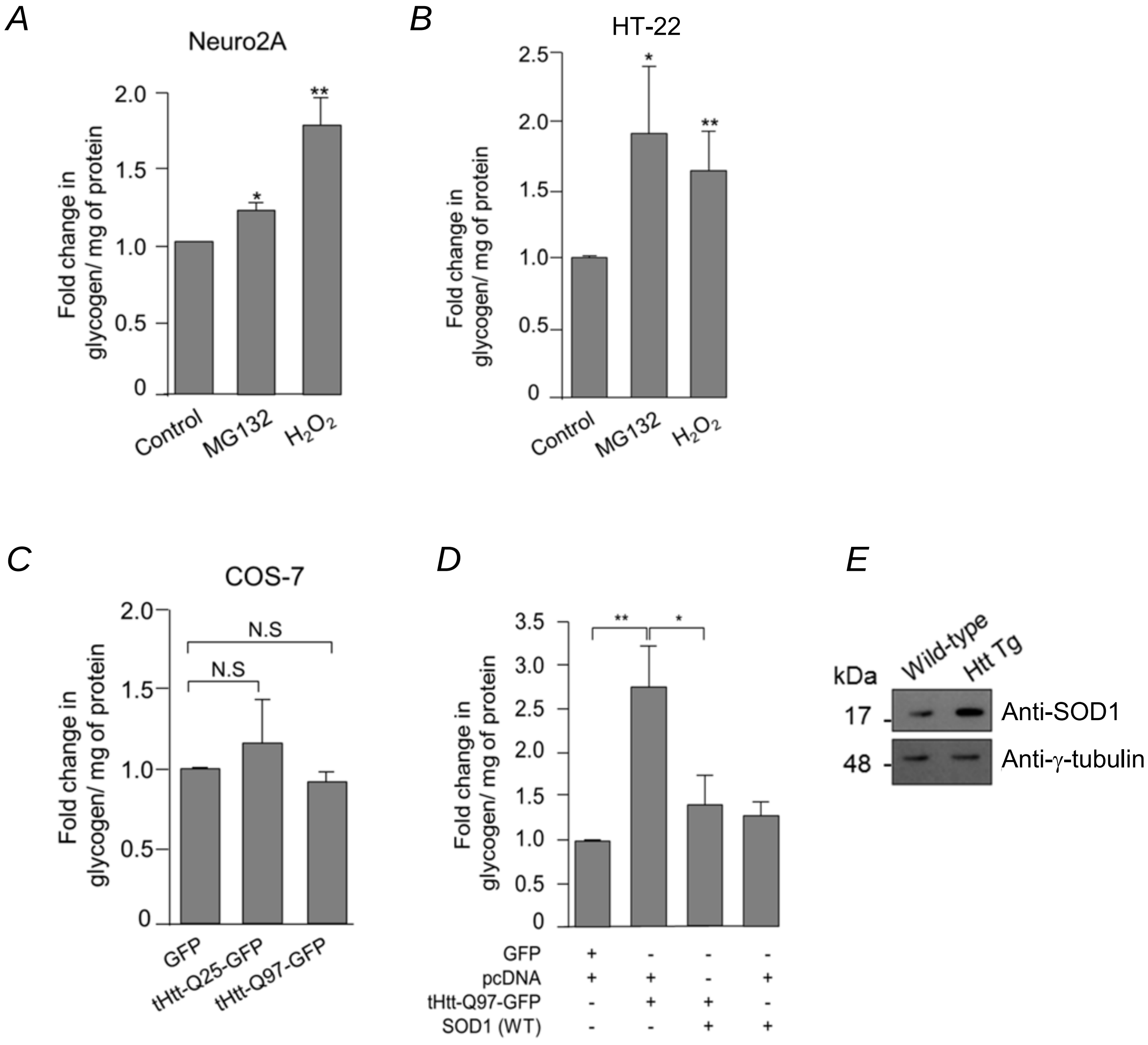

Supplementary Figure S1

Stress-induced glycogen synthesis in neuronal cells: (***A,B***) Bar diagram showing fold change in the glycogen level (normalized to protein content) upon exposure of Neuro2A (*A*) and HT-22 (*B*) cells to proteasomal inhibitor (10 μM MG132) or oxidative stress (1 mM of H_2O_2) for 6 Hrs. (N=3; t-test; *, $p \leq 0.05$; **, $p \leq 0.01$) (***C***) Bar graph showing the fold change in glycogen (normalized with protein content) in COS-7 cell line transiently over-expressed with tHtt-Q25-GFP and tHtt-Q97-GFP, as indicated. For calculating the change, the glycogen level measured in GFP-expressing cells was considered as 1 (N = 3; t-test; N.S., statistically not significant). (***D***) Bar diagram showing fold change in the glycogen level in Neuro2A cells transiently expressing the GFP or the tHtt-Q97-GFP either alone or along with the construct for the wild-type SOD1, as indicated (N=3; t-test; *, $p \leq 0.05$; **, $p \leq 0.01$). (***E***) Oxidative stress in the transgenic Huntington mouse brain. Representative immunoblot showing increased levels of SOD1 in brain tissues of the R6/2 line as compared to the age-matched wild-type line.

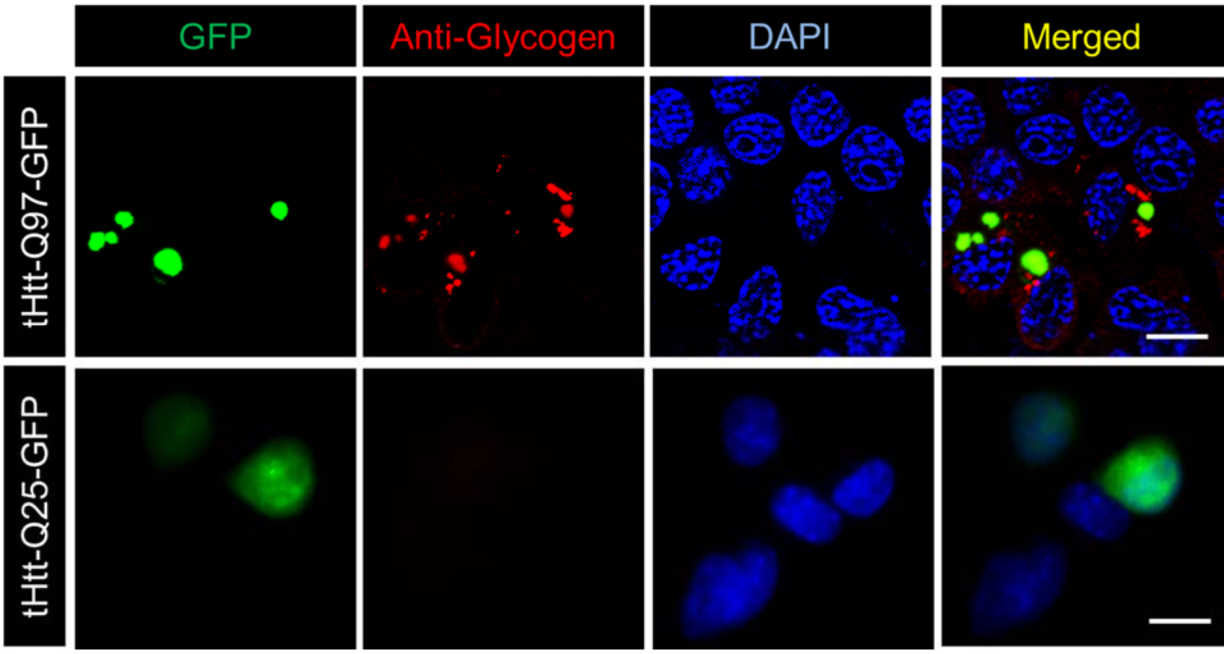

Supplementary Figure S2

Representative immunofluorescence images revealing glycogen granules (red color; stained using anti-glycogen antibody) in Neuro2A cells transiently expressing the tHtt-Q97-GFP (green signal) but not in the non-transfected cells or in cells that express the tHtt-Q25-GFP (green signal). The nuclei were stained using DAPI (blue). Scale bar, 10 μm.

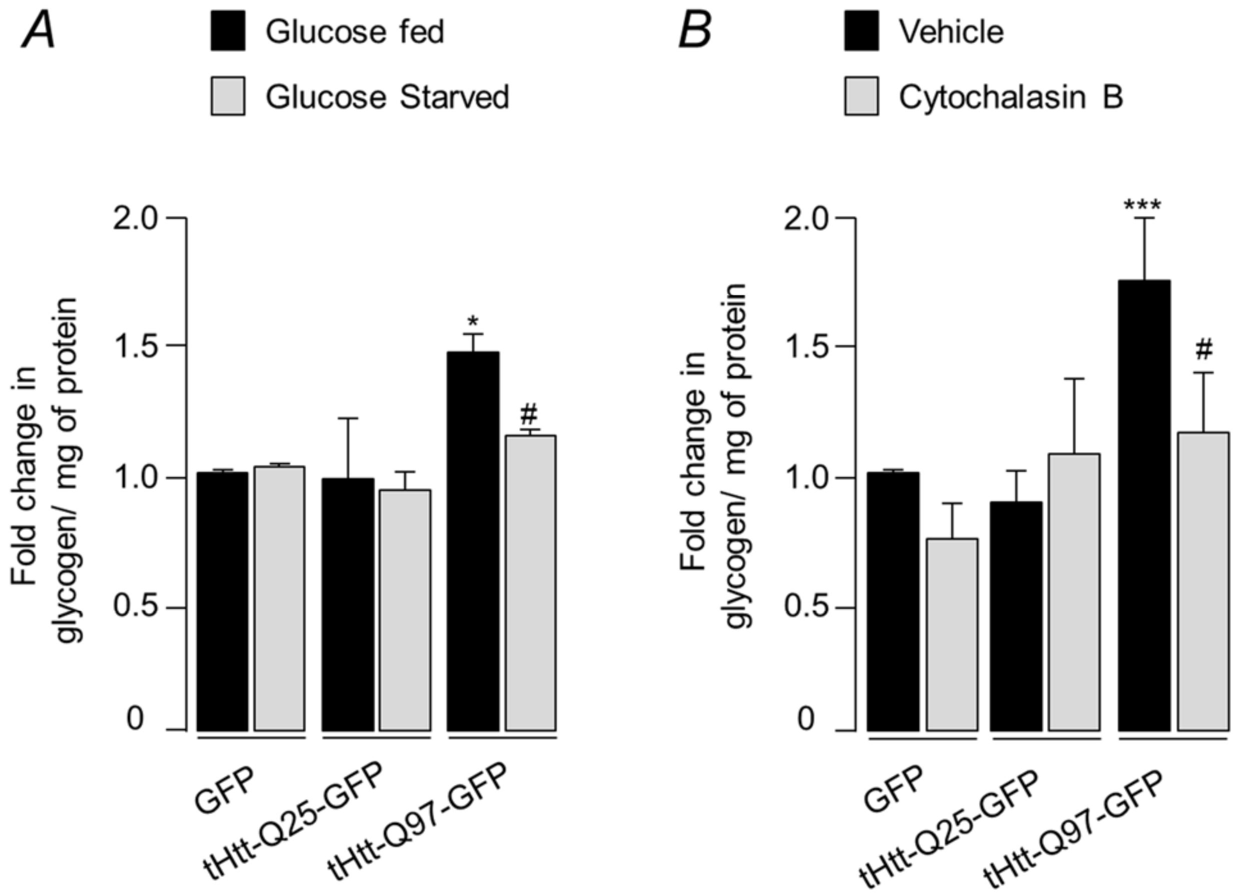

Supplementary Figure S3

The huntingtin-induced increase in neuronal glycogen level is due to its increased synthesis. (*A*) Bar diagram representing the fold change in the level of glycogen (normalized to protein) in Neuro2A cells transiently expressing the tHtt-Q25-GFP or tHtt-Q97-GFP when incubated in a glucose-rich medium (glucose fed) or in a medium lacking glucose (glucose starved for 6 hrs). The glycogen level in the GFP-expressing cells maintained in glucose-fed condition was considered as 1, and the relative levels in the other sets were plotted. (N = 3; t-test; * or # $p < 0.05$; * when compared with cells expressing GFP and glucose fed, and # when compared with cells expressing tHtt-Q97-GFP and glucose fed) **(*B*)** Bar diagram representing the fold change in glycogen in Neuro2A cells expressing tHtt-Q25-GFP or tHtt-Q97-GFP upon treatment with vehicle or Cytochalasin B (10 µM for 6 hrs). The glycogen level in the GFP-expressing cells treated with the vehicle was considered as 1, and the relative levels in the other sets were plotted. (N = 3; t-test; #, $p < 0.05$; ***, $p < 0.001$; * when compared with cells expressing GFP and vehicle treated, and # when compared with cells expressing tHtt-Q97-GFP and treated with vehicle).

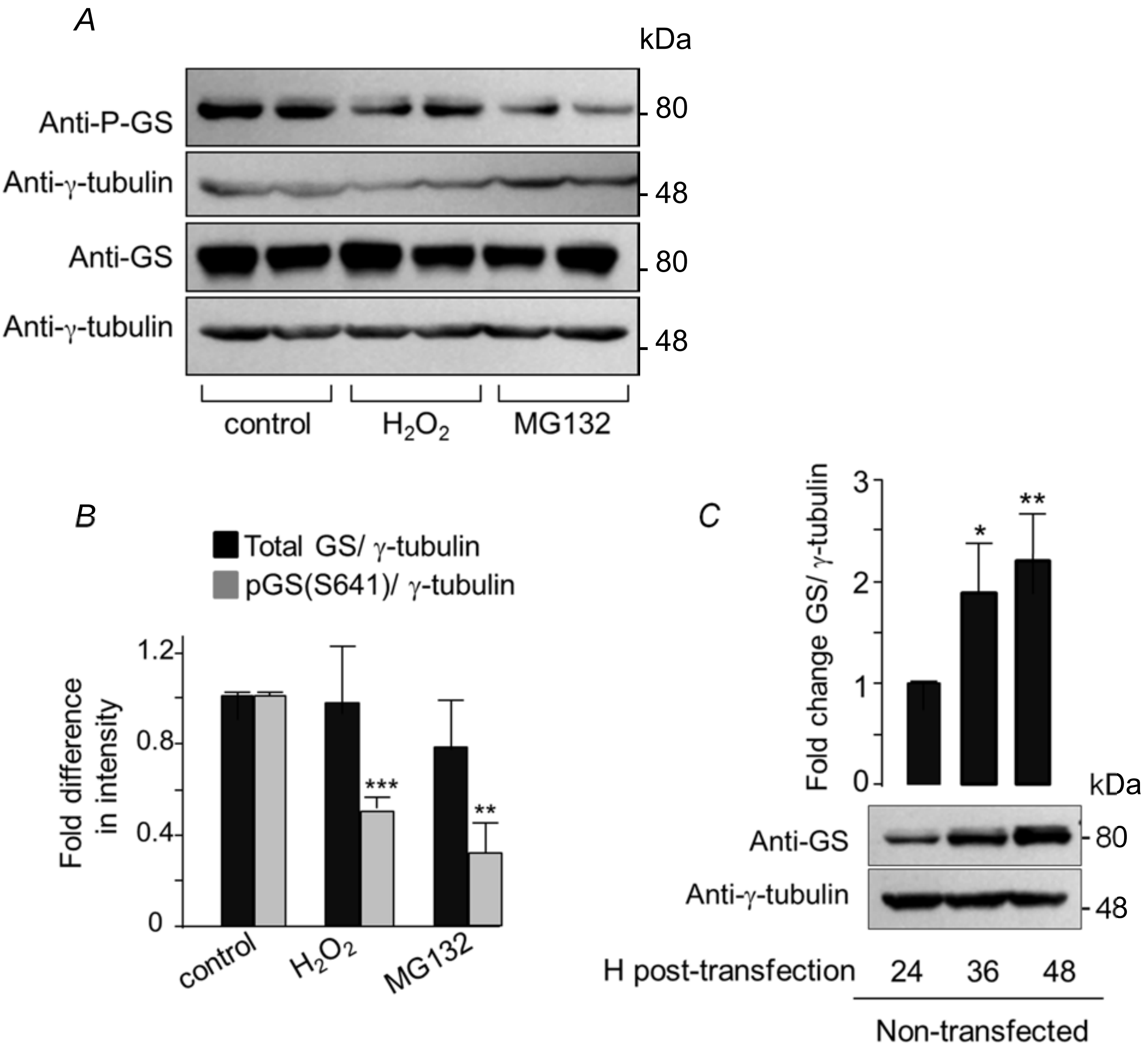

Supplementary Figure S4

(*A*) Representative immunoblots showing the relative levels of total GS and its phospho-S641 form in HT-22 cells exposed to oxidative (H_2O_2) and proteasomal (MG132) stress. Probing for γ-tubulin served as a loading control. **(*B*)** Bar diagram shows the fold changes in the signal intensities (measured by densiometric analysis of immunoblots as shown in (***A***) of the total GS and phospho-S641 GS, both normalized to γ-tubulin level. The signal intensities obtained for the untreated cells was considered as 1, the relative levels in the other set was plotted (N = 3; t-test; **, $p < 0.01$; ***, $p < 0.001$; *, compared with phospho-GS in untreated cells). **(*C*)** Representative immunoblots and densiometric analysis for GS levels in Neuro2A cells at indicated time points post-splitting. The values were normalized to the tubulin (loading control). The value obtained for the cells at 24 hrs post-splitting was considered as 1, and the rest were normalized accordingly (N=3; t-test; *, $p < 0.05$; **, $p < 0.01$).

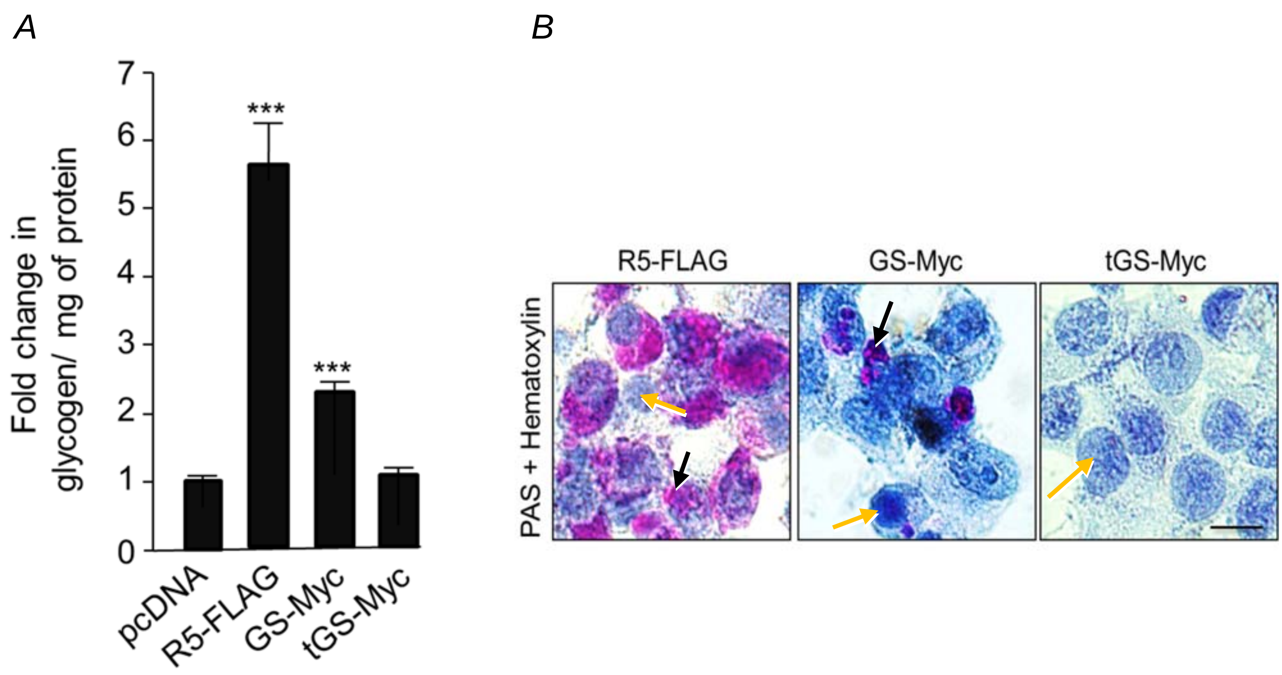

Supplementary Figure S5

(*A*) Bar diagram showing fold change in the glycogen level (normalized to protein content) in Neuro2A cells transiently expressing R5, GS, and truncated GS (tGS). The glycogen level in cells transfected with an empty vector (pcDNA) was considered as 1, and the relative levels in the other sets were plotted(N = 3; t-test; ***, p < 0.001). **(*B*)** PAS staining revealing increased glycogen (magenta color, indicated by black arrow) in Neuro2a cells transiently over-expressing R5, GS . Cells transfected with the construct that express the truncated GS (tGS) served as the control. The cells were counter-stained with hematoxylin (blue color, indicated by yellow arrow). Scale bar, 10 μm.

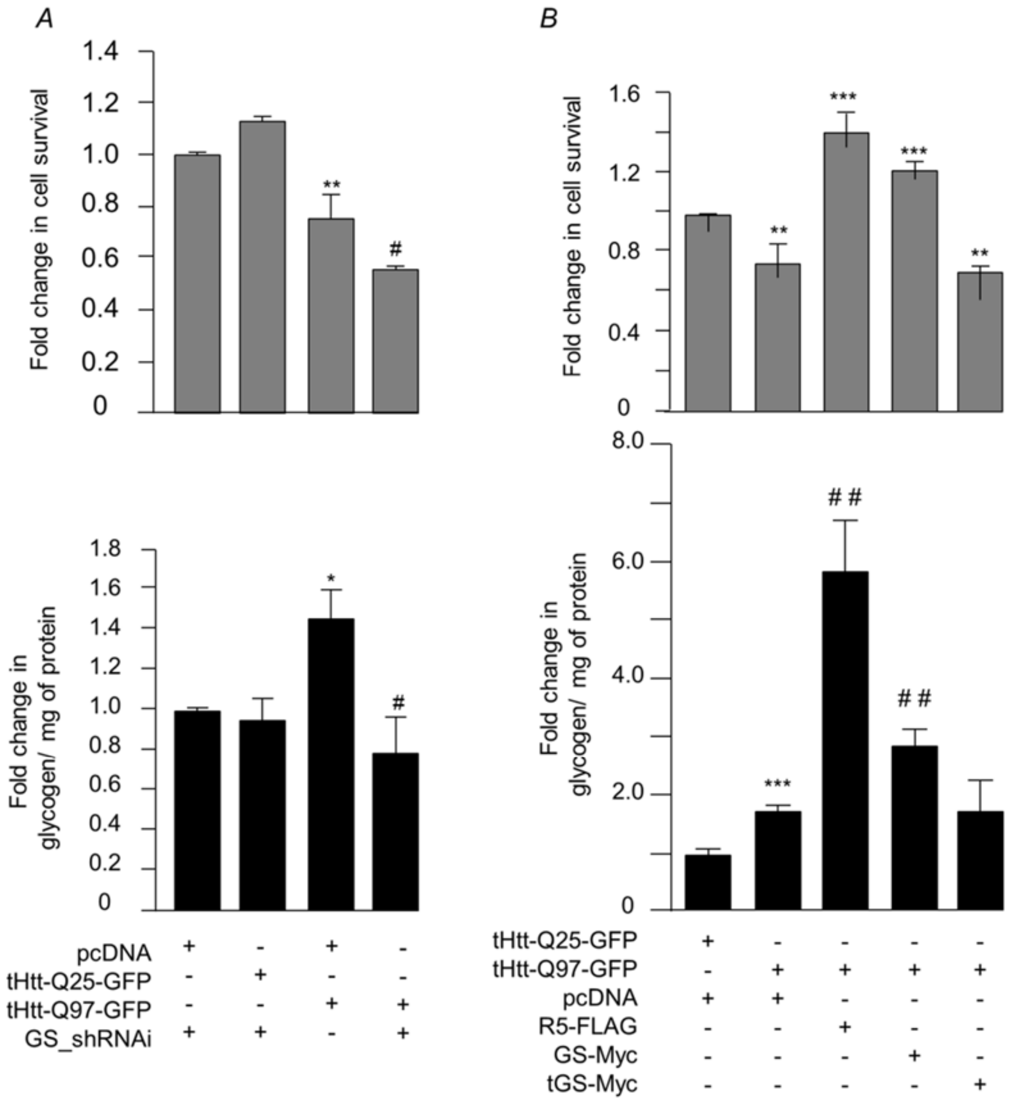

Supplementary Figure S6

Active glycogen synthase protects Neuro2A cells from the mutant huntingtin-induced cytotoxicity: (*A*) Bar diagram showing fold change in survival and glycogen levels (normalized to protein) of Neuro2A cells expressing GS_shRNAi, tHtt-Q25-GFP or tHtt-Q97-GFP – either alone or in combination, as indicated. The survival rate for the cells transfected with the GS_shRNAi was considered as 1, and the relative levels in the other sets were plotted. Similarly, glycogen level of cells expressing GS_shRNAi was considered as 1, and relative levels in other sets were plotted (N = 3; t-test; * or $^{\#}$ $p \leq 0.05$; **, $p \leq 0.01$; * , compared with cells co-expressing pcDNA and GS_shRNAi, and # when compared with cells co-expressing tHtt-Q97-GFP and pcDNA). **(*B*)** Bar diagram showing fold change in survival and glycogen levels (normalized with protein) of Neuro2A cells transiently expressing tHtt-Q25-GFP, tHtt-Q97-GFP, R5-FLAG, GS-Myc or truncated GS-Myc – either alone or in combination as indicated. The survival rate was measured using MTT assay and for the cells expressing tHtt-Q25-GFP was considered as 1, the relative levels in the other sets were plotted. Similarly, glycogen levels in cells expressing tHtt-Q25-GFP was considered as 1, and the relative levels in the other sets were plotted (N = 3; t-test; **or $^{\#\#}$, $p \leq 0.01$; *** , $p \leq 0.001$; *, compared with tHtt-Q25-GFP expressing cells; $^{\#}$, compared with tHtt-Q97-GFP expressing cells).

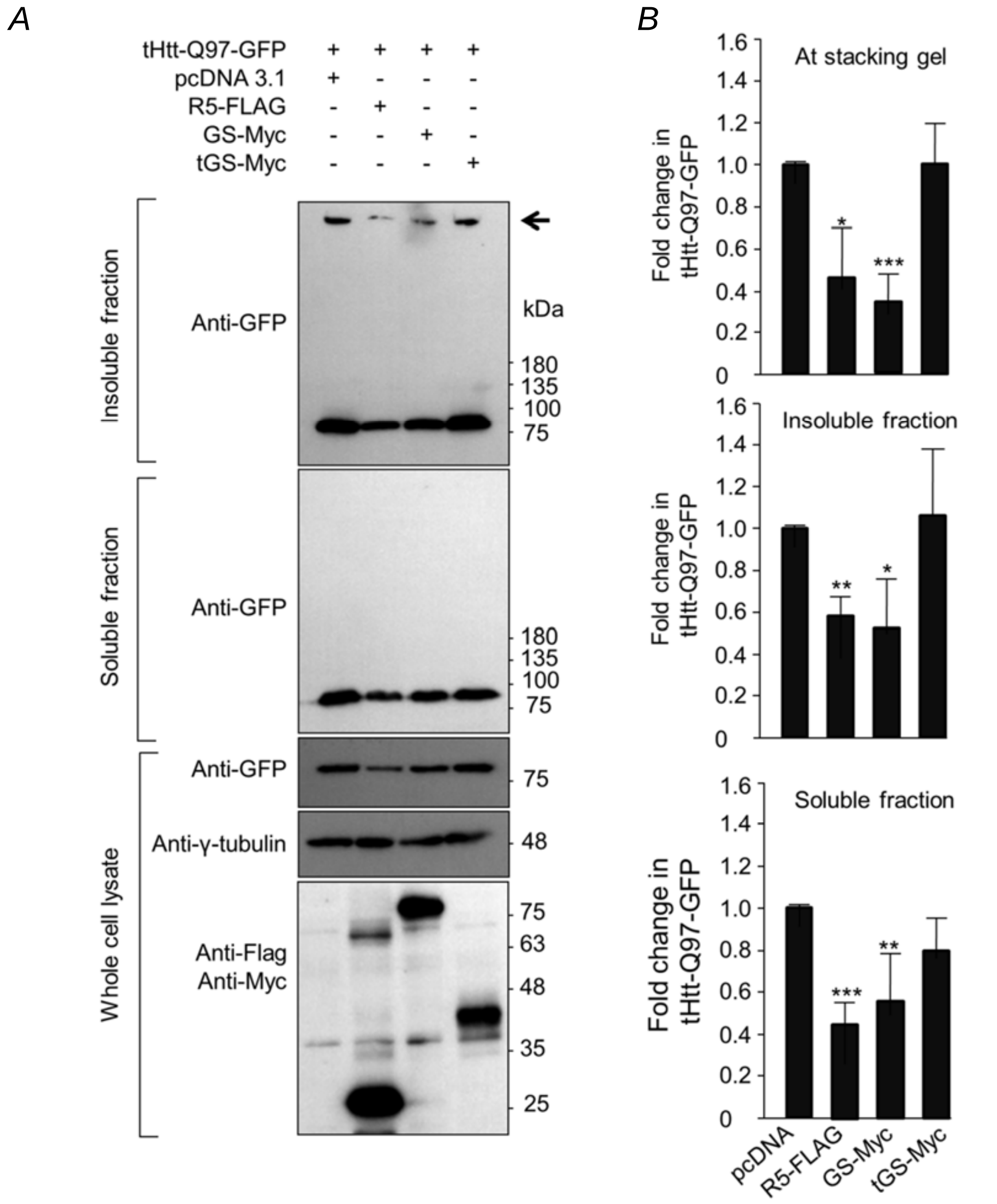

Supplementary Figure S7

Increased glycogen reduces the level of insoluble, aggregated form of huntingtin in Neuro2A cells: **(*A*)** Representative immunoblot showing the relative levels of mutant huntingtin (tHtt-Q97-GFP) in Neuro2A cells transiently coexpressing the empty vector (pcDNA), R5, GS or the truncated GS (tGS), as indicated, in the detergent (NP-40) soluble or insoluble fraction. Arrow identify the aggregated huntingtin trapped in the stacking gel. The relative levels the transiently expressed protein in the whole cell lysate are also shown. **(*B*)** Bar diagram showing the relative levels (signal intensities) of mutant huntingtin in the stacking gel, insoluble, or the soluble fraction, as indicated. For all, the values were normalized to the tubulin (loading control). The value obtained for the cells expressing the empty vector (pcDNA) was considered as 1, and the rest were normalized accordingly. For statistical significance, the values were compared with the value obtained for the empty vector (N=3; t-test; *, $p \leq 0.05$; **, $p \leq 0.01$; ***, $p \leq 0.001$).

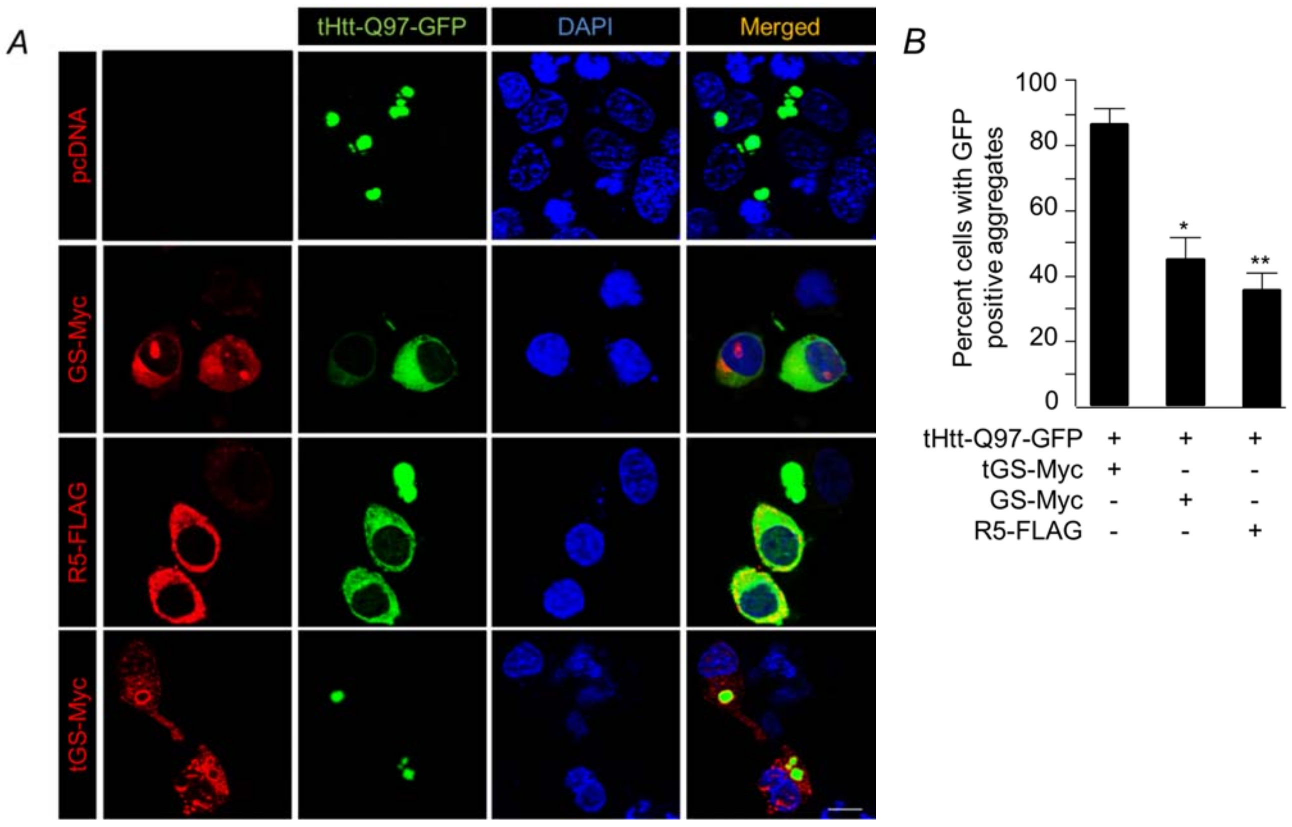

Supplementary Figure S8

Glycogen synthase level inversely correlate with the aggregation of mutant huntingtin in Neuro2A cells: (*A*) Representative immunofluorescence images of Neuro2A cells transiently coexpressing tHtt-Q97-GFP with R5, GS or the truncated GS (tGS), as indicated. Cells transfected with the empty vector (pcDNA) served as control. Nuclei were stained using DAPI (blue). Note the absence of GFP-positive aggregates in cells coexpressing R5 or GS. Scale bar, 10 µm. **(*B*)** Bar diagram showing frequency of Neuro2A cells positive for huntingtin aggregates when coexpressed with R5 or GS. For statistical analyses, these values were compared with the value obtained for cells coexpressing the truncated GS (tGS) (N = 3; t-test; *, $p \leq 0.05$; **, $p \leq 0.01$).

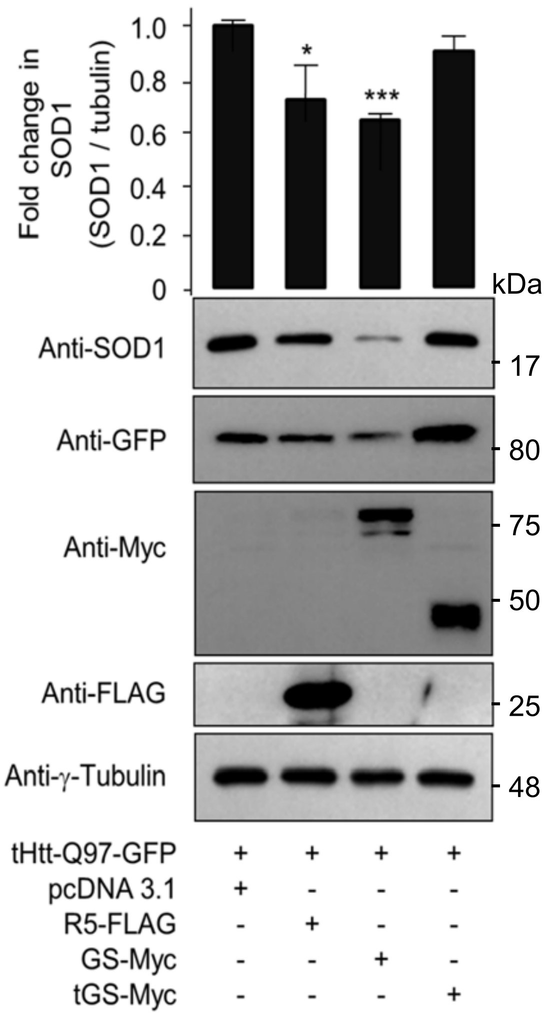

Supplementary Figure S9

Increased glycogen level ameliorates mutant huntingtin-induced oxidative stress in Neuro2A cells. Representative immunoblots and densiometric analysis for SOD1 levels in cells transiently coexpressing tHtt-Q97-GFP with either the empty vector (pcDNA), R5, GS or the truncated GS (tGS). For all the values were normalized to the tubulin (loading control). The value obtained for the cells expressing the empty vector (pcDNA) was considered as 1, and the rest were normalized accordingly. For statistical significance, the values were compared with the value obtained for the empty vector (N=3; t-test; *, $p \leq 0.05$; ***, $p \leq 0.001$).

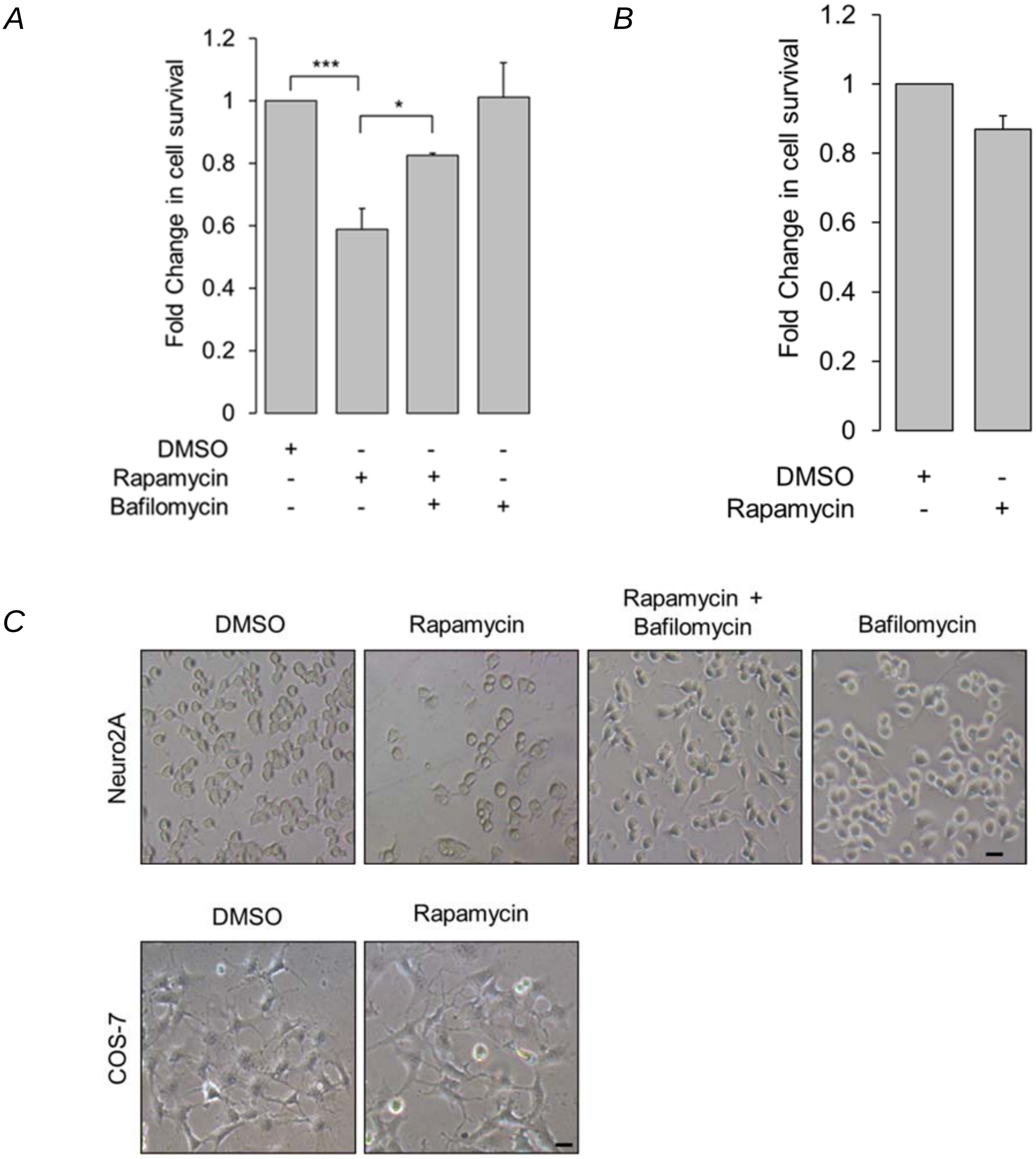

Supplementary Figure S10

Enhanced autophagy flux is deleterious for neuronal cells: (*A*) Bar diagram showing fold change in survival of Neuro2A cells treated with rapamycin (autophagy inducer), either alone or with bafilomycin (autophagy blocker) and the cell survival was measured by MTT assays. Values obtained for cells treated with the vehicle (DMSO) was considered as 1, and the fold change in the survival for the other treatments was plotted. (N=3; t-test and One-way ANOVA Dunnett's multiple comparison testing; *, $p \leq 0.05$; ***, $p \leq 0.001$). **(*B*)** Bar diagram showing the change in the survival of COS-7 cells treated with rapamycin, as measured by MTT assay. **(*C*)** Representative phase-contrast micrograph showing the morphology of Neuro2A or COS-7 cells treated with the indicated drug. Note the restoration of confluence and morphology of Neuro2A upon double treatment of with rapamycin and bafilomycin. Scale bar, 10 µm.

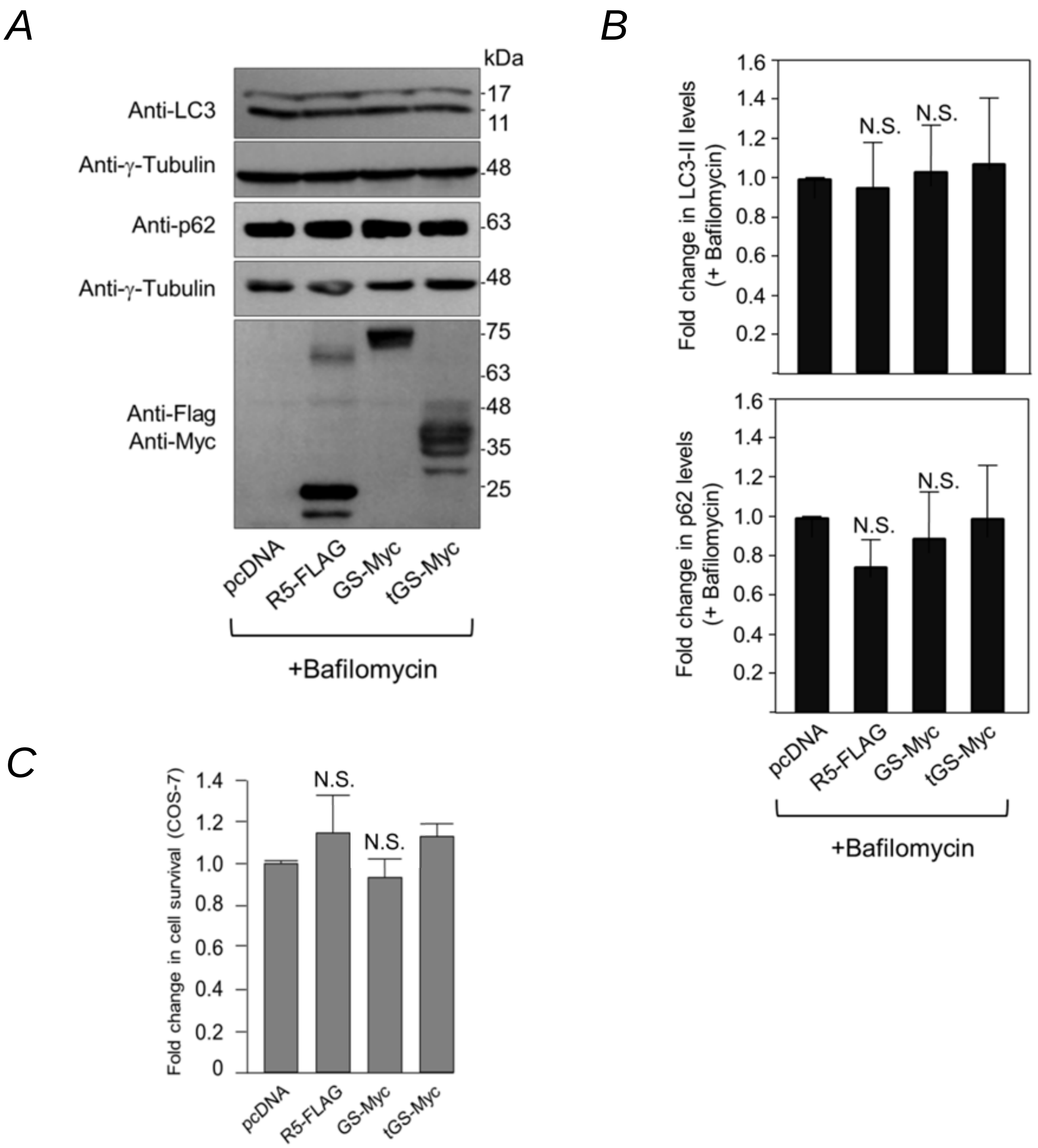

Supplementary Figure S11

(***A***) Representative immunoblots showing the relative levels of LC3 and p62 in Neuro2A cells transiently overexpressing R5, GS, the truncated GS (tGS) or the empty vector (pcDNA) in the presence of bafilomycin (an autophagic blocker). Probing for γ-tubulin served as the loading control, and probing for FLAG/MYC epitope confirmed the expression of the constructs. (***B***) Bar diagram, shows the fold changes in the signal intensities (measured by densiometric analysis) of the p62 or the LC3-II, both normalized to γ-tubulin level. The signal intensities obtained for p62 and LC3-II for the control set [empty vector (pcDNA) transfected cells] was considered as 1, the relative levels in the other sets were plotted (N = 3; t-test; N.S is statistically not significant). (***C***) Over-expressed GS and R5 are toxic only in neuronal cells. Bar diagram showing the fold change in the survival of COS-7 cells transiently expressing R5, GS or the truncated GS, as measured by MTT assays. Values were normalized to cells transfected with an empty vector (pcDNA). (N = 3; t-test; N.S is statistically not significant).

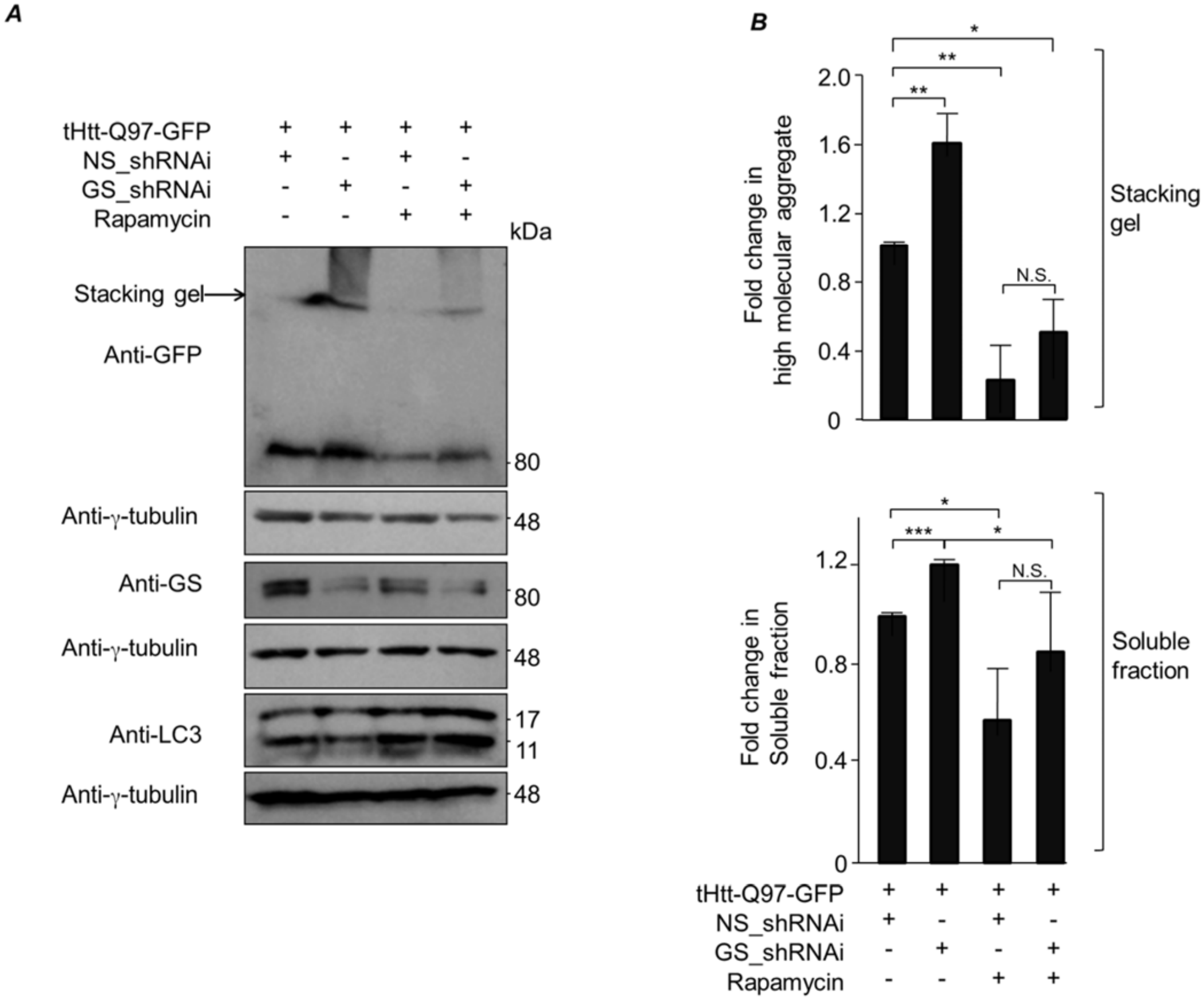

Supplementary Figure S12

Cellular glycogen might modulate the aggregation kinetics of mutant huntingtin. (*A*) Representative immunoblot showing the presence of insoluble form tHTT-Q97-GFP in stacking gel and in soluble form in resolving gel when the cells partially silenced for DS (GS_shRNAi) and treated or not treated for the autophagic inducer, rapamycin. Cells transfected with the non-silencing construct (NS_shRNAi) served as control and probing for γ-tubulin served as a loading control. **(*B*)** Bar diagram showing the fold changes in the signal intensities (measured by densiometric analysis) of soluble and insoluble form of mutant huntingtin seen in Fig A. Signal intensity obtained from the cells lysates that expressed the mutant huntingtin (lane 1) was considered as 1, and the relative levels in other sets are plotted (N=3; t-test and one-way ANOVA Dunnett's multiple comparison testing ; *, $p<0.05$; **, $p < 0.01$; ***, $p < 0.001$; N.S is statistically not significant).

Supplementary Table S1:

Table showing age, sex and post-mortem intervals (wherever available) of the brain tissues used for the histochemical analyses. The clinical diagnosis of the subjects was made by trained neurologists. The age matched control samples represent subjects who were neurologically normal but succumbed to road traffic accidents.

Category	Clinical Diagnosis (sample ID)	Age / sex	Post-mortem interval
Affected	Alzheimer's Disease (A12/80)	83 y / Male	Not available
	Parkinson's Disease (97/B41)	62 y/ Male	2 hr 40 min
	Pick's Disease (97/B42)	Not available	Not available
Normal	Control 1 (07/B189)	55 y / Male	7 hr
	Control 2 (13/B254)	53 y /Male	9 hr
